# Dendritic cell-derived exosomes induce monocyte antigen-presentation and immune amplification in neoantigen vaccine therapy

**DOI:** 10.3389/fimmu.2025.1565696

**Published:** 2025-05-19

**Authors:** Shinji Morisaki, Hideya Onishi, Takafumi Morisaki, Makoto Kubo, Masayo Umebayashi, Hiroto Tanaka, Norihiro Koya, Shinichiro Nakagawa, Kenta Tsujimura, Sachiko Yoshimura, Poh Yin Yew, Kazuma Kiyotani, Yusuke Nakamura, Masafumi Nakamura, Takehiro Torisu, Takanari Kitazono, Takashi Morisaki

**Affiliations:** ^1^ Fukuoka General Cancer Clinic, Fukuoka, Japan; ^2^ Department of Medicine and Clinical Science, Graduate School of Medical Sciences, Kyushu University, Fukuoka, Japan; ^3^ Department of Surgery and Oncology, Graduate School of Medical Sciences, Kyushu University, Fukuoka, Japan; ^4^ Cancer Precision Medicine Inc., Kanagawa, Japan; ^5^ National Institutes of Biomedical Innovation, Health and Nutrition, Osaka, Japan

**Keywords:** dexosomes, neoantigen, neoepitope, dendritic cell, vaccine, immune amplification

## Abstract

Mature dendritic cells release exosomes; however, the immunological role of exosomes in dendritic cell vaccine therapy remains unclear. We examined the immunogenicity of neoantigen peptide-pulsed dendritic cell-derived exosomes (Neo-P DEX) and investigated their role in vaccine therapy. The quality of DEX derived from dendritic cell cultures was confirmed via electron microscopy, western blotting, flow cytometry, and CD63 ELISA. When DEX released from neoantigen-pulsed DCs was applied to monocytes, they showed dendritic cell-like properties such as surface antigen expression. Furthermore, monocytes receiving Neo-P DEX activated neoantigen-reactive T lymphocytes. Fluorescence-activated cell sorting (FACS) analysis showed that plasma exosomes after neoantigen-pulsed DC vaccine may contain more DEX compared to before the vaccine, suggesting that DEX released after DC vaccination may be involved in the amplification of tumor-specific immune responses by translocating to monocytes in the patient body and transforming them into antigen-presenting dendritic cells. This study suggests that dendritic cell exosomes may act as endogenous neoantigen vaccines or immune amplifiers.

## 1 Introduction

The importance of the adaptive immune system against cancer and highly infectious diseases has been demonstrated in recent years by the efficacy of immune checkpoint inhibitors in cancer therapy and the preventive effects of the mRNA vaccine against coronavirus disease 2019 (COVID-19) (1–5). Activation and proliferation of antigen-reactive naïve T lymphocytes by antigen-presenting cells such as dendritic cells (DCs) are essential for activating the adaptive immune system against cancer and pathogens (6, 7).

Vaccine therapy using DCs that present cancer antigens has been widely reported for its safety and efficacy (8, 9). Neoantigens, newly generated in cancer cells due to genetic mutations, have recently garnered particular attention among cancer antigens presented by DCs (10) and tumor specificity (11, 12). Indeed, there have been many recent reports on the safety and efficacy of neoantigen vaccine therapy (13–15). Moreover, numerous reports highlight that neoantigens are utilized in vaccines and play a role in the effectiveness of immune checkpoint inhibitors (16, 17). Furthermore, we have previously reported the efficacy of neoantigen vaccine therapy using antigen peptide-impregnated monocyte-derived DCs (18–21). In our dendritic vaccine therapy approach, DCs pulsed with neoantigen peptides are administered into lymph nodes, as detailed in a recent paper (22). Recently, we reported on the immunological analysis of DC vaccines pulsed with Class II affinity long peptide containing a Class I neoantigen epitope (23).

Nevertheless, the details of the immune response that occurs *in vivo* when a DC vaccine is administered to a patient remain unclear. The ELISpot response demonstrates that administration of the neoantigen-pulsed DC vaccine results in the activation of neoantigen-reactive naïve T lymphocytes, which are increased in the peripheral blood after the vaccine. The direct activation of T lymphocytes takes place in the lymph node. The influx of antigen-reactive T lymphocytes into the lymph node represents the most probable mechanism of immune response when mature DCs, pulsed with antigen, are introduced into the lymph node, as described in our method (22). However, mature DCs are known to release dexosomes (DC-derived exosomes: DEX); in mouse models and *in vitro* experiments, DEX has been shown to have the same antigen and co-stimulatory molecules as those of DCs and that they can stimulate other antigenic T cells (24–30). In 1998, Zitvogel et al. reported the anti-tumor effect of DEX in a mouse mode (31), and its clinical efficacy in humans has since been investigated; however, the effect is currently limited (32–34).

However, the physiological role of DEX in DC vaccine therapy remains unclear. Specifically, it is uncertain whether DEX released from neoantigen-pulsed DCs can enhance the vaccine’s effect by fusing with other antigen-presenting cells. We hypothesized that administration of the neoantigen peptide DC vaccine would result in the local release of DEX, which would then migrate locally or systemically to other antigen-presenting cells, such as monocytes, which could serve as neoantigen-presenting DCs.

In patients undergoing neoantigen peptide-pulsed DC vaccine therapy, culture supernatants collected from the DC vaccine used in therapy, peripheral blood mononuclear cells, and plasma before, during, and after treatment are cryopreserved for monitoring immune responses. **Supplementary Figure 1** illustrates the protocol for intranodal DC therapy and DEX collection and preservation. In this study, we examined the effect of DEX derived from neoantigen-pulsed DC culture supernatant on unstimulated monocytes in patients whose immune response to neoantigen was increased by this treatment. The patients analyzed for DEX encompassed three individuals who were administered the Class-I and Class-II hybrid neoantigen peptide-pulse DC vaccine therapy, as we recently reported (23). We found that incorporating DEX into monocytes derived from the patient’s peripheral blood induced the monocytes to become functional DC-like cells that present neoantigens. In other words, DEX released from DCs administered into lymph nodes migrates systemically and is taken up by non-activated antigen-presenting cells such as monocytes and transformed into antigen-presenting cells, which may be involved in the immune amplification of the DC Vaccine. A diagram regarding the overall view of this study is illustrated in **Supplementary Figure 2**.

## 2 Materials and methods

### 2.1 Ethics approval

Ethics Committee of Fukuoka General Cancer Clinic (FGCC EC009) approval and written consent from the patients were obtained in accordance with the Act on Safety Assurance of Regenerative Medicine in Japan.

### 2.2 Patients

Cryopreserved PBMCs and peptide-pulsed DC culture supernatant-derived exosomes before and after treatment were used after explanation and written consent of seven patients undergoing neoantigen peptide-pulsed DC vaccine therapy. The seven patients in the immunological analysis of DEX in this study all had advanced solid tumors with Stage IV metastases and were treated with Neoantigen Peptide DC Vaccine tumor therapy in combination with chemotherapy and had an immune response to Neoantigen (**Supplementary Figure 6**, **Table 1**). The types of tumors were esophageal cancer (1 case), colorectal cancer (1 case), ovarian cancer (3 cases), sarcoma (fibrous tumor) (1 case), and pancreatic cancer (1 case), and Flowchart of the simplified treatment process is shown in **Supplementary Figure 7**. **Supplementary Table 2** shows the Class-I affinity Neoantigen Peptide and Class-II affinity Neoantigen Peptide selected for each patient. Inclusion criteria include patients who underwent neoantigen-pulsed DC vaccine therapy during the study period, demonstrated a strong immune response, and consented to provide blood and other samples. Exclusion criteria encompass patients who fail to meet any of these criteria. DC vaccine protocol, neoantigen analysis, and patient immune cell analysis were similar to those described in previous reports (18–21, 23, 35, 36), and Ethics Committee of Fukuoka General Cancer Clinic approval and written consent from the patients were obtained in accordance with the Act on Safety Assurance of Regenerative Medicine in Japan. **Table 2** lists the clinical profiles of the seven patients who underwent DEX analysis. Exosomes isolated from pre- and post-treatment plasma, post-treatment PBMC, and DC culture medium supernatants, collected from patients at leukapheresis before and after the DC vaccine administration, were cryopreserved and used for the immunological analysis of DEX.

**Table 1:** Profiles of neoantigens and immunological responses.

#Patient	Number of SNV	Number of neoantigen	Number of selected neoantigen (short)	Number of selected neoantigen (long)	Number of ELISpot- positive peptide (short)	Number of ELISpot- positive peptide (long)
1	76	281	7	5	5	3
2	32	134	2	1	1	1
3	149	364	2	2	1	1
4	17	33	3	3	3	3
5	25	24	3	3	3	0
6	28	72	5	(–)	1	(–)
7	46	115	5	4	0	1

**Table 2:** Clinical profiles of the study participants.

Patient No.	Sex/Age	Tumor	Metastasis	Stage	Sample for neoantigen analysis (fresh or FFPE)	Prior therapy	Combined therapy	Initial Response	Overall Survival (month)
**1**	M/73	Esophagus	Liver and LNs	IV	Fresh (origin tumor)	Chemotherapy	Check Point Inhibitor	PR	36
**2**	F/48	Ovarian	Peritoneum	IV	Fresh (peritoneum)	Surgery and chemotherapy	CBDCA + Paclitaxel	CR	79
**3**	M/69	Colon	Liver and peritoneum	IV	FFPE (Origin tumor)	Surgery and chemotherapy	FOLFOX	PR	54
**4**	F/52	Sarcoma	Lung, liver, and bone	IV	FFPE (Liver metastasis)	Surgery, chemotherapy, and radiation	Pazopanib, Eribulin	SD	121
**5**	F/36	Ovarian	Liver and peritoneum	IV	FFPE (Origin tumor)	Surgery, chemotherapy, and radiation?	CBCDA+PTX	PR	31
**6**	F/62	Ovarian	peritoneum	IV	FFPE (Origin tumor)	Surgery	PARP inhibitor	CR	54
**7**	M/73	pancreas	LN	IV	FFPE (Origin tumor)	Surgery	GEM/nPTX	PR	56

FFPE, Formalin-Fixed; Paraffin-Embedded; GEM, Gemcitabine; LN, Lymph Node; LNs, Lymph Nodes; nPTX, Nab-Paclitaxel; PARP, Poly (ADP-Ribose) Polymerase; PD, Progressive Disease; PR, Partial Response; SD, Stable Disease.

### 2.3 DC vaccine culture and administration

The DC vaccine was cultured as previously reported (18). Briefly, PBMCs collected and cryopreserved in leukapheresis before treatment were thawed and seeded in DC complete medium containing 1% autologous serum in 6 well plates (FALCON, Franklin Lake, NJ, USA) for 30 min (2 × 10^6^/mL). After removing floating cells and washing twice with Roswell Park Memorial Institute medium, adherent cells were cultured in a DC complete medium containing 100 ng/mL of granulocyte-macrophage colony-stimulating factor (Primmune Inc., Kobe, Japan) and 50 ng/mL of IL-4 (Primmune). On day 6 of culture, maturation factors (MFs) containing 500 IU/mL of TNF-α (PeproTech Inc., Rocky Hill, NJ, USA) and 500 IU/mL of IFN-α (Sumitomo Pharma, Osaka, Japan) were added. Neoantigen Peptide was dissolved in sterile water containing dimethyl sulfoxide before use, filtered through a 0.22-μm Millipore syringe (Millipore, Mosheim, France), and finally tested for endotoxin, β-glucan, and mycoplasma to ensure that they were below detection limits. Endotoxin and β-glucan were analyzed using a Toxinometer ET-600 (Wako Pure Chemical Industries, Osaka, Japan). Mycoplasma contamination was detected using MycoAlert (Lonza Rockland Inc., Rockland, ME, USA). Class I short peptides were added following the introduction of MF, whereas Class II long peptides were incorporated prior to the MF addition. The supernatants from DC cultures were collected and preserved at 4°C for subsequent exosome isolation, and DEX extraction was performed as detailed below. Prior to the collection of mDCs, tests for endotoxin, β-glucan, and mycoplasma contamination in the culture medium indicated levels below detection limits. FACS analysis confirmed the DCs were positive for HLA-DR, HLA class I, CD86, and CD40 but negative for CD14. This analysis utilized the Navios EX system (Beckman Coulter, Inc., Brea, CA, USA). Cell counting was conducted using the Sysmex CD-500 system (Sysmex Inc., Kobe, Japan). DCs were resuspended in saline (0.5 mL), loaded into a 1 mL syringe fitted with a 25 G needle, and administered under ultrasound guidance by an experienced physician, targeting the cortical-medullary junction of normal inguinal lymph nodes. This technique has been previously described (18).

### 2.4 Separation and extraction of exosomes

Extraction of exosomes from DC culture supernatants or plasma was performed by stepwise ultracentrifugation. The refrigerated DC culture supernatant was centrifuged (1640 g 15 min, 4°C), and the supernatant was filtered through a 0.22 μm filter. The filtrate was transferred to an ultracentrifuge tube and centrifuged (100,000 × *g*, 70 min 4°C) in an OptimaTM MAX-TL Ultracentrifuge (Beckman Coulter. Inc.). The supernatant was aspirated, resuspended in filtered PBS (–), collected in a single ultracentrifuge tube, and ultracentrifuged at 100,000 × *g* for 70 min at 4°C. The supernatant was then aspirated again, resuspended in filtered PBS (–), and the concentration was determined using the CD63 exosome ELISA Kit before being frozen at -80°C in low-protein adsorption tubes until use. For the separation of the exosome fraction from plasma, plasma stored at -80°C was thawed, centrifuged at 10,000 × *g* for 10 min at 4°C, filtered through a 0.22 μm filter, transferred to an ultracentrifuge tube, and ultracentrifuged at 100,000 × *g*. Exosome extraction was carried out using the ExoQuick-TC kit (System Biosciences, Palo Alto, CA, USA) as per the manufacturer’s instructions. The isolated exosomes were resuspended in filtered PBS (–), and after measuring the concentration with the CD63 exosome ELISA Kit, Human (Hakarel Inc., Ibaraki, Japan), a portion was stored at -80°C in a low-protein adsorption tube.

### 2.5 Western blotting

To the exosome solution, 4× loading dye (2-ME-free) and PBS were added as necessary, and the mixture was incubated in a heat block at 37°C for 30 min. Subsequently, SDS polyacrylamide gel electrophoresis was conducted using a gradient gel (XV, 40 mA). Protein transfer from the gradient gel to the PDVF membrane was carried out, followed by immersion of the membrane in a blocking solution, which was blocked for 1 h at room temperature. The membrane was then incubated with a solution of either anti-CD9 antibody (clone ALB6, Santa Cruz Biotechnology #sc-59140) or CD63 antibody (clone H5C6, BD Bioscience #556019), each diluted 200-fold in 10% Blocking One/TBST. Following this, the membrane was treated with HRP-conjugated anti-mouse IgG antibody (secondary antibody) diluted 2,000 times in 10% BlockingOne/TBST and added to the container holding the membrane, where it was incubated for 45 min with shaking. ChemilumiOne (Nakaraitesk Corp.) was utilized to detect chemiluminescence. The raw western blot data are included separately in **Supplementary Figure 8**.

### 2.6 Electron microscopy

Exosome morphology was examined using electron microscopy (149-EX31) at FujiFilm Wako Jyunyaku (Osaka, Japan) and photographed (JEOL JEM1400 flash at 100 kV). Particle size was measured using a nanoparticle tracking method (NanoSight LM10).

### 2.7 Confocal laser microscopy

Exosomes were extracted from the culture supernatant of DCs cultured from patient-derived monocytes by stepwise ultracentrifugation. The extracted exosomes were fluorescently labeled with Exostep and used in the following experiments.

For the isolation of CD14+ monocytes, PBMCs were harvested from the patient’s apheresis, and CD14+ monocytes were subsequently separated with a 25% yield using human CD14 microbeads (Miltenyi Biotec) at a concentration range of 0.5–0.6 M (1 × 10^6^). Fluorescently labeled exosomes and CD14+ monocytes were then co-cultured in a 1 ml tube at room temperature for 3 h, followed by centrifugation at 1,500 rpm for 8 min. The supernatant was removed, the pellet was gently tapped, and 8 μL of FITC mouse anti-human CD14 antibody was added. This mixture was incubated at room temperature for 30–50 min, after which the supernatant was aspirated, and the pellet was again gently tapped. The cells were washed with immunostaining buffer, centrifuged at 1,500 rpm for 8 min, and the supernatant was discarded. The pellet was resuspended in 250 μL of immunostaining buffer per well and seeded onto observation plates. DAPI was added, and cells were examined under a confocal laser microscope (LSM700) using 20x, 40x, and 63x oil immersion lenses (exostep flour dye: red 555 nm, FITC: green 488 nm, DAPI: blue 445 nm).

### 2.8 Observation of uptake into CD14+ monocytes after the addition of DEX

Exosomes and CD14+ monocytes were prepared using the same procedure as previously described. A total of 0.25 M (1 × 10^6^) CD14+ monocytes were seeded in 24-well plates. Observation of DEX uptake into monocytes was conducted by fluorescence inverted microscopy (OLYMPUS CORPORATION Inverted Microscope IX71) 24 h after the addition of DEX.

### 2.9 Surface antigen analysis of DEX and DEX-added monocytes

Surface antigen analysis of DEX and plasma-derived exosomes was conducted using anti-CD9 antibody-conjugated exosome-capturing microbeads (Exo StepTM, Human; Immunostep S.L., Salamanca, Spain), adhering to the manufacturer’s guidelines. DEX or plasma-derived exosome suspensions (2 ng/mL) underwent interaction with CD9 antibody-bound microbeads for 2 h at 37°C, after which the expression of CD40, CD86, and HLA-DR on the bead surface was assessed via FACS. In a separate experiment, DEX or plasma-derived exosomes were adsorbed onto monocytes, and their surface antigen expression (CD40, CD86, and HLA-DR) was evaluated using FACS.

### 2.10 ELISpot reaction

ELISpot analysis was performed using the Human IFN-γ ELISpotplus kit (MABTECH, Cincinnati, OH, USA) according to the manufacturer’s instructions. Briefly, 96-well plates were washed four times with sterile PBS (200 μL) and pretreated with medium containing 10% autologous serum (200 μL) used for cell suspension for 30 min–1 h, then the medium was removed, and immune cells, peptides, or exosomes were added using the following assay method. After a 48-h incubation, the plate was washed five times with 200 μL of PBS. Subsequently, the detection antibody (7-B6-1-biotin) was dissolved in PBS containing 0.5% fetal bovine serum (PBS-0.5% FCS) at a concentration of 1 μg/mL. The resulting solution (100 μL/well) was added, and the plate was incubated at approximately 15–25°C for 2 h. Following this, the plate was washed five times with 200 μL of PBS, and the secondary antibody (Streptavidin-HRP) was prepared in PBS-0.5% FCS at a dilution of 1:1,000. This antibody solution (100 μL/well) was added and incubated for 1 h at approximately 15–25°C. After incubation, the plate was washed five times with 200 μL of PBS to remove any residual solution, and 100 μL/well of TMB substrate solution was added and allowed to react for 10 min. To halt the reaction, the plate was washed five times with deionized water, the wells were dried, and the spots were detected and analyzed using an automated ELISpot reader 0.8 classics (AID GmbH, Strasberg, Germany).

### 2.11 Mixed culture of Class-I/II hybrid neoantigen long peptide-pulsed DC-derived exosomes with peripheral blood mononuclear cells

DCs were pulsed with Class-I/II hybrid neoantigen peptide (25 μg/mL), after which maturation factors were added. Subsequently, culture supernatants of DCs were collected after an additional 48 h, and exosomes were extracted as previously described. As controls, DEX obtained without peptide addition was also extracted. In two patients (Pts. 1 and 3), monocytes isolated using CD14-positive selective beads were cultured at 5 × 10^3^ cells/well in ELISpot plates. DEX was then added at a concentration of 2 ng/mL and cocultured for 24 h. Subsequently, CD8+ T cells isolated from PBMCs obtained after three cycles of neoantigen DC vaccine therapy using CD8+ T-positive selection beads were added to the ELISpot plates at 2 × 10^5^ cells/well and cultured for 48 h to evaluate the ELISpot response.

In a separate experiment, immature DCs from Pt. 1 and Pt. 3 were cultured (5 × 10^3^ cells/well in 96-well ELISpot plates) with DEX for 24 h. A maturation cocktail was added and gently washed twice with the culture medium. Subsequently, isolated CD8+ T lymphocytes (2 × 10^5^ cells/well) were added and cultured for 48 h to facilitate the IFN-γ ELISpot reaction.

### 2.12 Characterization and functional analysis of plasma exosomes

Plasma exosomes from the patients were extracted from 2 mL of cryopreserved plasma before and after 3 vaccine doses and measured using CD63 ELISA. The surface antigen analysis of these exosomes was conducted through FACS analysis using CD9-supplemented beads, as previously described. In some experiments, ELISPOT reactions were performed using PBMCs from Pts. 1 and 3 with or without the plasma exosomes at a concentration of 1 ng/mL. The ELISPOT reaction procedure was detailed in previous studies.

### 2.13 Time-dependent/quantity-dependent analysis of DEX of immune response by mixed culture of DC-derived exosome-added monocytes + lymphocytes

In one patient (Pt. 1), CD14 monocytes were isolated using CD14+ positive selection beads and cultured in ELISpot plates at a density of 5 × 10^4^ cells/well. DEX (0.4 ng/well) was added after 24 and 48 h. The ELISpot reaction was assessed by adding 2 × 10^5^ lymphocytes per well to the ELISpot plate after 24 and 48 h and incubating for 72 h. Control groups included monocyte alone, lymphocyte alone, and a combination of monocyte and lymphocyte. In a subsequent experiment, 5 × 10^3^ cells/well of isolated monocytes were cultured in ELISpot plates, and DEX (1.4 ng/well) was added in dilutions of 1x, 0.1x, 0.01x, and 0.001x. After 24 h, 2 × 10^5^ cells/well of lymphocytes were added to the ELISpot wells and incubated for 48 h to measure the ELISpot reaction. Control groups were monocytes, lymphocytes, a combination of monocytes and lymphocytes, and lymphocytes treated with DEX.

### 2.14 Measurement of NFκB p65 in the nucleus of monocytes added with DEX

Monocytes isolated from one patient (Pt. 1) using CD14+ positive selection beads were spread on six-well plates, DEX was added, and after 24 h, cells on the plates were collected, and nucleoproteins were extracted according to the protocol (RayBiotech Nuclear Extraction Kit (50 extractions). Control was nucleoprotein extracted from monocytes without DEX and absorbance of NFκB p65 was measured according to the protocol (Invitrogen NFκB p65 (total/Phospho) Human 85 -86083–11 InstantOne ELISA kit: 96 tests).

### 2.15 Detection of intracellular cytokines in monocytes with DEX

In two patients (Pt. 1 and 7), immature DCs were prepared, pulsed with neoantigen peptides, and exosomes were extracted as previously described (Material and methods 2.4). CD14+ monocytes (Mo) were isolated from apheresis-derived PBMCs using CD14+ positive selection beads. These monocytes were then cultured in 6-well plates, and DEX was added, whereas PBS was used in the control group. After 24 h, cells were harvested from the plates, a protein transport inhibitor was added, and 5 h later, staining with CD14-FITC (30 min) and IL12-PE (30 min) was conducted following fixation/permeabilization treatment. An anti-human IL-12 antibody was utilized. FACS analysis was subsequently carried out. The permeabilization process employed the BD Cytofix/Cytoperm Plus Fixation/Permeabilization kit (BD, Cat. No. 554715).

### 2.16 Gene expression of DEX-added monocytes

RNA was extracted from monocytes isolated from cryopreserved PMBCs of Pt.1 using anti-CD14mAb beads and from monocytes collected 12 h after the addition of Pt. 1 DEX using the AllPrep DNA/RNA mini kit (Qiagen Inc., Venlo, Netherlands). RNA-seq libraries were prepared using the TruSeq Standard mRNA Library Prep kit (Illumina Inc., San Diego, CA, USA). The prepared RNA-seq libraries were prepared using TruSeq Standard mRNA Library Prep kit (Illumina Inc.). The RNA-seq data were analyzed using Cufflinks software41 and FPKM (per kilobase of transcript per million mapped reads) values.

### 2.17 Statistical analysis

Statistical analyses were performed using GraphPad Prism 8 (GraphPad Software Inc., San Diego, CA, USA). Data are presented as mean ± standard deviation (SD). Student’s t-test was used to compare continuous variables between the two groups. Statistical significance was set at *p* < 0.05. Experiments were performed in triplicate.

## 3 Results

### 3.1 Characterization of DC-derived exosomes

**Figure 1:**
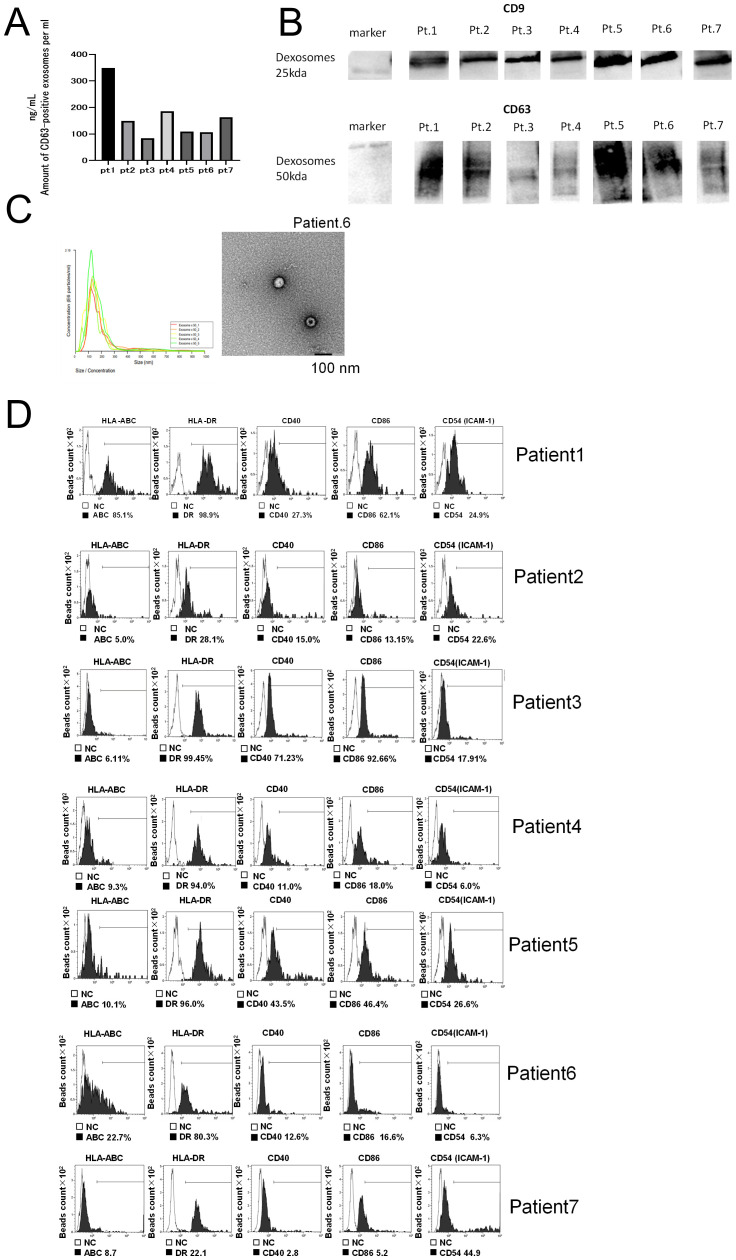
Quality analysis of dexosomes (DEX). **(A)** DEX concentration from mature DCs (Pts 1–7) pulsed with neoantigen-peptide (CD63 ELISA); the concentration of CD63+ exosomes extracted from 10 mL DC culture supernatant was measured after suspension in 200 μL PBS. **(B)** Western blot analysis of DEX (neoantigen-peptide pulsed DC culture supernatant derived from Pts 1 to 7). **(C)** Electron micrograph of DEX derived from Pt 6. **(D)** Dexosome FACS analysis; 2 ng of DEX derived from Pts 1 to 7 were bound to CD9 capture microbeads and analyzed for HLA class I, HLA-DR, CD40, CD86, and ICAM-1 by FACS analysis (white in each panel indicates the isotype control for each antibody). DC, Dendritic Cell; DEX, Dendritic Cell-Derived Exosomes; PCR, Polymerase Chain Reaction; INF, interferon; Pt, patient.

We analyzed the characteristics of extracellular vesicles (ECVs) extracted from the culture supernatant of each patient’s matured DC (50 mL total). We first measured the concentration of CD63, a marker of exosomes. The CD63 concentration of ECVs obtained from each patient was detected in the range of 100–300 ng/mL (CD63 ELISA) (**Figure 1A**). To confirm that these were DC-derived exosomes (DEX), we performed western blot analysis, which detected CD9 as well as CD63 protein expression, confirming that they were indeed DEX (**Figure 1B**). In addition, the DEX derived from Pt 6 was examined using electron microscopy, and the nanoparticle tracking method confirmed that it consisted of vesicles approximately 100 nm in size (**Figure 1C**). To confirm the presence of surface antigen molecules on the membrane vesicles released from DCs pulsed with neoantigen peptides, supernatants from DC cultures of Pts 1–7 were adsorbed onto anti-CD9-coated microbeads for 12 h. FACS examination revealed surface expression of HLA-A, B, and C; CD40; CD86; HLA-DR; ICAM-1, confirming DEX (**Figure 1D**).

### 3.2 Analysis of DEX translocation to monocytes

**Figure 2:**
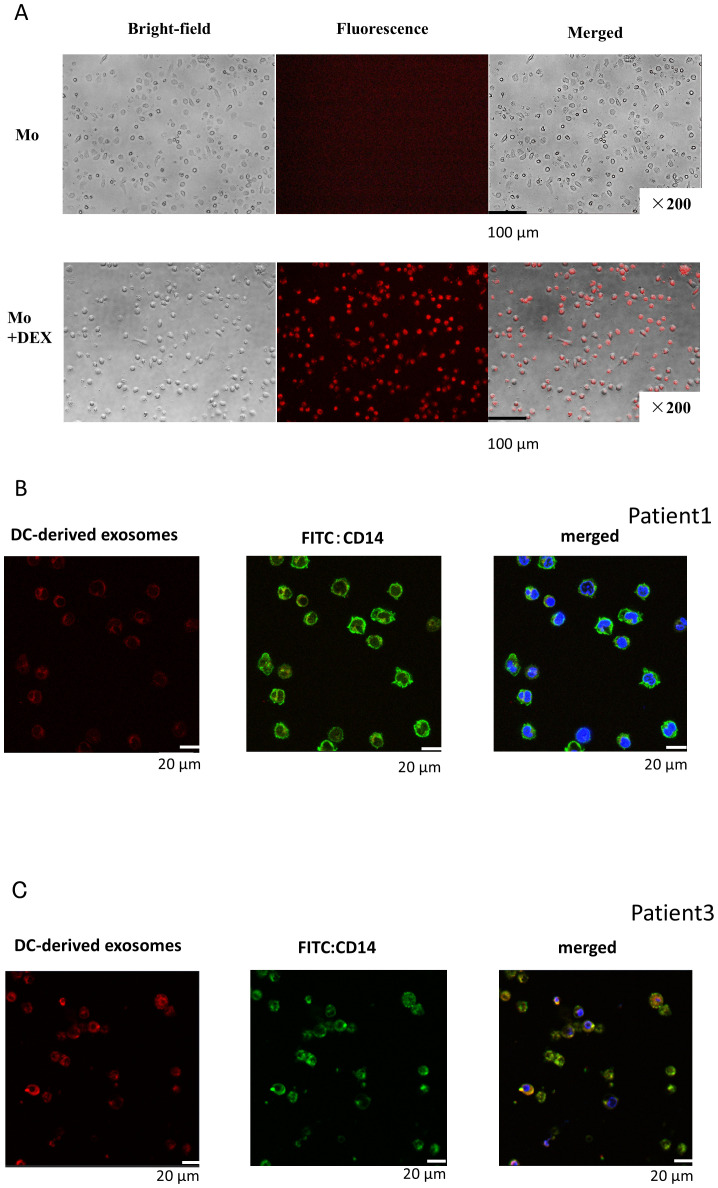
DEX uptake into monocytes. **(A)** DEX uptake into monocytes (fusion image of fluorescently stained DEX in monocytes). Evaluation of DEX uptake into monocytes by fluorescence microscopy. DEX from Pt 1 was fluorescently labeled, the unbound dye was removed, and the labeled DEX were incubated with monocytes derived from the patient peripheral blood for 24 h and observed under a fluorescence microscope. Most monocytes were stained red, indicating that DEX were bound to or incorporated into the cells. **(B, C)** Images of migration of fluorescently labeled DEX within monocytes by confocal microscopy: Composite image of fluorescently labeled DEX (red, left) and CD14 antibody-fluorescently labeled monocytes (green, center) with nuclei stained blue with DAPI (right). Pt, patient.

**Figure 3:**
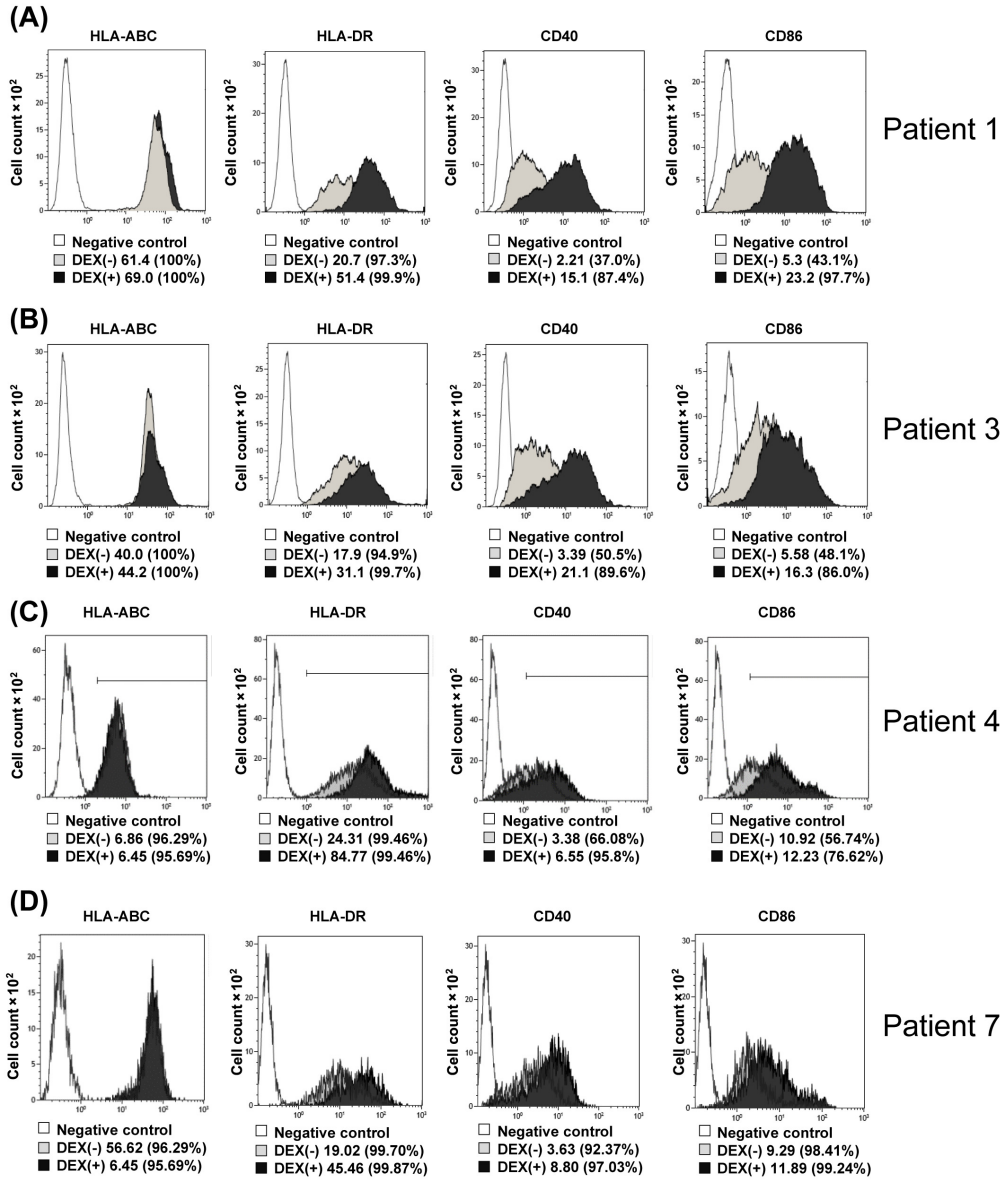
Changes in surface antigens of monocytes upon addition of DEX. **(A–D)** DEX (2 ng/mL) from Pts 1, 3, 4, and 7 were added to CD14+ selected monocytes from the same patients and incubated for 24 h, followed by FACS analysis of HLA-Class-I, HLA-DR, CD40, and CD86 expression (**A–D**, respectively). Pt, patient; DEX, Dendritic Cell-Derived Exosomes.

DEX released from neoantigen peptide-pulsed DCs could be taken up by other antigen-presenting cells (APCs) to present neoantigen to CD8T lymphocytes. Therefore, we first examined whether DEX can be taken up by resting monocytes. When fluorescently labeled DEX was added to monocytes, these cells exhibited fluorescence 24 h later (**Figure 2A**: Top figure without DEX, bottom figure with addition of DEX). To further examine the incorporation of DEX into monocytes, fluorescence-labeled autologous DEX was added to CD14+ monocytes derived from Pt. 1 (**Figure 2B**) and Pt. 3 (**Figure 3C**). Intracellular uptake of DEX was observed 3 h later using confocal laser microscopy (**Figures 2B, C**; red DEX, blue: nuclei, green: CD14+Mo).

### 3.3 Changes in surface antigens of DEX-added monocytes

Next, changes in monocyte surface antigen by adding DEX to monocytes were examined using Pt1, 3, 4, and 7 samples. As shown in **Figures 3A–D** (A; Pt. 1 data, B; Pt. 3 data, C; Pt. 4 data, D; Pt. 7 data), when DEX (0.5 ng/mL) was added to monocytes isolated with anti-CD14 antibody-attached beads and incubated for 12 h, the surface antigens on monocytes with CD40, CD86, and HLA-DR were significantly higher than those of the control (monocytes to which DEX were not added). These results indicate that DEX-loaded monocytes change to dendritic-like cells in surface antigens.

### 3.4 Examination of the ability of DEX-loaded monocytes to present neoantigen antigen

**Figure 4:**
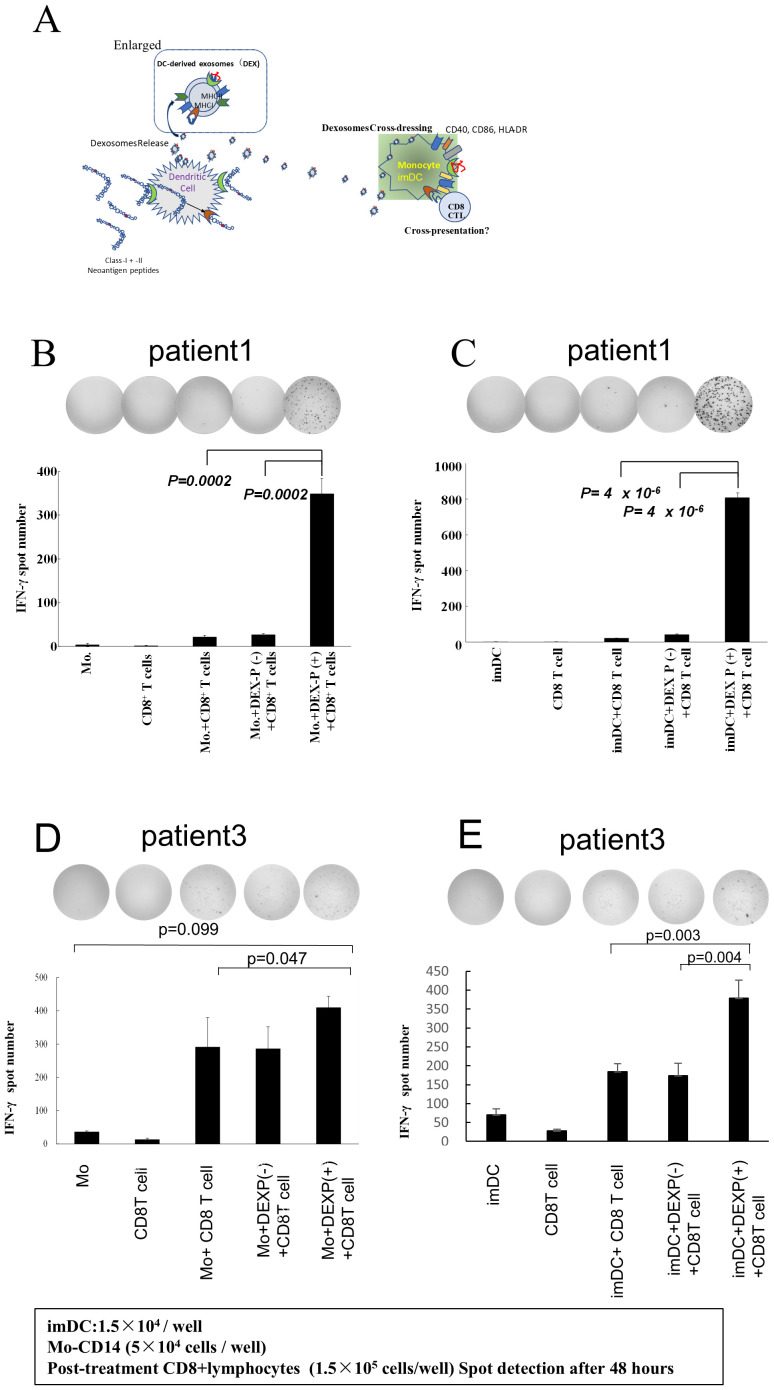
DEX-added monocytes or immature DCs activate neoantigen-reactive CD8T cells. **(A)** Experimental schema. **(B, D)** Activation of antigen-reactive CD8 T lymphocytes by DEX-loaded monocytes from Pts 1 and 3: DEX was added to their respective monocytes, and CD8 T cells from PBMC collected and cryopreserved after three vaccinations were co-cultured with the monocytes. The monocytes were cultured for 48 h to assess ELISpot responses. Controls included monocytes alone, CD8+ T cells alone, and CD8 T cells reacted with monocytes pulsed with peptide without DEX (b, data from Pt 1; d, data from Pt 3). Notably, only monocytes crossed with neoantigen peptide-pulsed DEX-induced stimulation of CD8+ T cell responses. **(C, E)** DEX-added monocyte-derived imDCs, along with CD8 T lymphocytes, significantly activated CD8 T lymphocytes in the group treated with neoantigen-added DC-derived exosomes. Pt, patient; DEX, Dendritic Cell-Derived Exosomes.

We determined whether DEX derived from neoantigen peptide-pulsed DC could be transferred to monocytes and activate them to present antigen to antigen-reactive T lymphocytes. Pts 1 and 3 correspond to the cases we detailed in our earlier report concerning the immunological and clinical effects of the Neoantigen peptide-pulsed DC vaccine (23). In this study, we specifically analyze the immunological impact of DEX on autologous monocytes in these patients. Furthermore, we investigated whether DEX extracted from Pts. 1 and 3 neoantigen-peptide-pulsed DCs could stimulate class I affinity antigen-responsive CD8+ T cells by adding 2 ng/ml to monocytes. **Figure 4A** shows the hypothesis schema of this experiment. As illustrated in **Figures 4B, D** (B; data from Pt. 1, D; data from Pt. 3), CD8+ T cells isolated from PBMC following neo-DC vaccination demonstrated a significant (p<0.05) immune response to neoantigen-DEX-added monocytes, whereas the response of CD8+ T cells to control peptide (–) DEX-added monocytes or to monocytes alone was relatively small.

Similarly, when neoantigen peptide-pulsed DC-derived DEX was added to immature DCs, CD8+ T cells showed strong interferon (IFN)-γ production response (**Figures 4C, E**; C; data from Pt. 1, E; data from Pt. 3). These results suggest that DEX-containing class II hybrid long peptides were taken up and processed by monocytes to acquire the capability to present class I neoantigen peptides. Furthermore, when DEX was added to monocytes, the response of T lymphocytes was evaluated under varying amounts and durations; it was found to increase in a dose-dependent manner (**Supplementary Figure 3A**). Additionally, the timing of DEX addition to monocytes, whether at 24 or 48 h, did not alter the outcome, indicating that antigen presentation by DEX-treated monocytes peaked at 24 h (**Supplementary Figure 3B**).

### 3.5 Changes in intracellular protein and gene expression caused by DEX transfer into monocytes

Next, we examined how DEX uptake into monocytes induces activation signals and gene expression in monocytes. First, we examined changes in the p65 nuclear protein in Pt.1 DEX-exposed monocytes. The results showed that DEX exposure increased nuclear p65 in monocytes, suggesting nuclear factor kappa-light-chain-enhancer of activated B cells (NF-κB) activation (**Supplementary Figure 4A**). Second, we examined whether DEX exposure affected interleukin (IL)-12 production in monocytes. The results indicated that IL-12 production is elevated in DEX-exposed monocytes from Pt.1 and Pt.7 (**Supplementary Figure 4B**). Subsequently, to assess changes in immune-related mRNA expression, we extracted mRNA from monocytes treated with and without DEX, comparing the expression of various immune-related genes. Notably, we observed increased mRNA expression of several chemokine receptors, IL-2R, TNF, and HLA-E (**Supplementary Table 1**).

### 3.6 Investigation of monocyte activation *in vivo*

**Figure 5:**
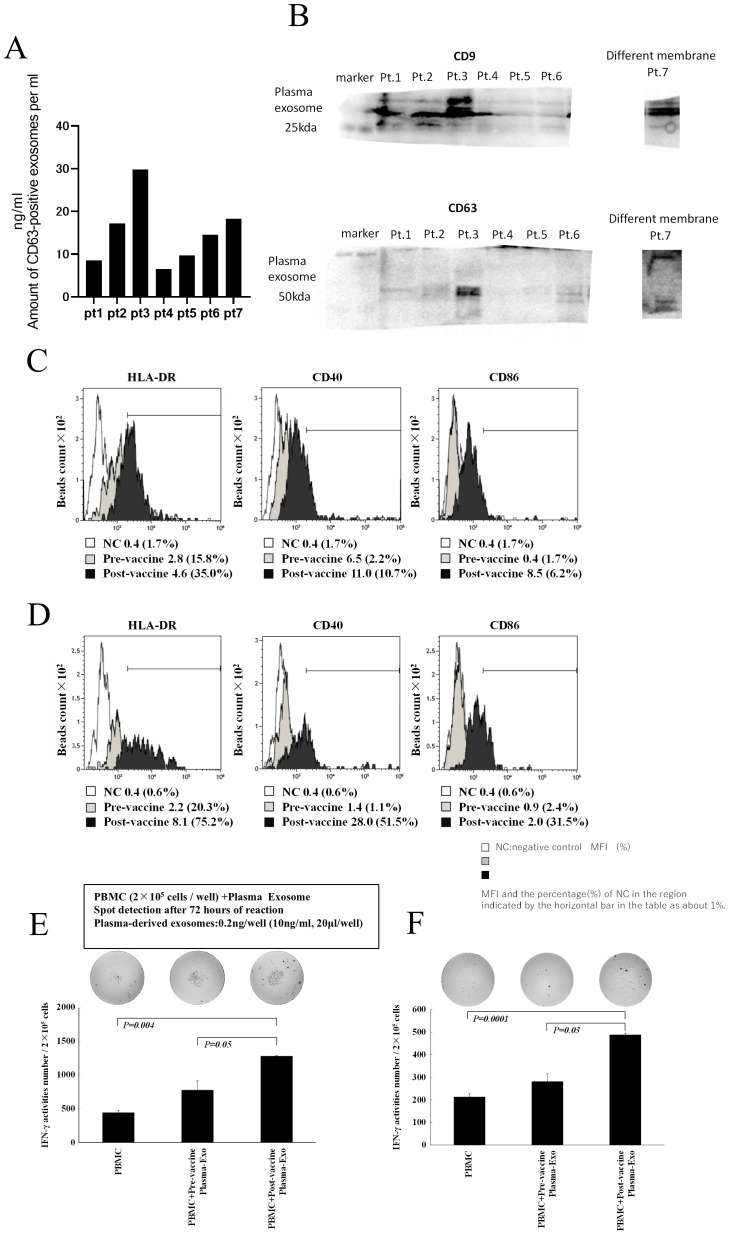
Characterization of plasma exosomes following administration of neoantigen peptide-pulsed DC vaccine. **(A)** Western blot analysis of plasma exosomes (top: CD9, bottom: CD63, Pts1–7). **(B)** Plasma exosome concentration: exosomes were extracted from 10 mL of plasma and suspended in 200 μL PBS, and their concentration was measured by CD63 ELISA. **(C, D)** Surface antigen analysis of plasma exosomes (HLA-A, B, C, HLA-DR, CD40, and CD86). Plasma exosomes after vaccination showed increased positivity and relative intensity of expression of HLA-DR, CD40, and CD86 compared with plasma exosomes before vaccination (**C**, data from Pt 1; **D**, data from Pt 5). The negative control was isotype-matched fluorescent-labeled nonspecific antibody binding. **(E, F)** Activation of T lymphocytes by exosomes in plasma after vaccine administration: Treatment with plasma exosomes obtained after vaccination in Pt 1 **(E)** and Pt 5 **(F)** increased IFN-γ production in PBMCs. Pt, patient.

To investigate the possibility that DEX released from DCs by intranodal administration of DC vaccine could activate monocytes *in vivo*, we thawed monocytes that had been cryopreserved before and after the DC Vaccine, added lymphocytes after vaccine sensitization and examined their activation using the ELISpot assay. As shown in **Supplementary Figure 5**, in 6 cases, post-vaccine peripheral blood monocytes tended to activate more lymphocytes than pre-vaccine monocytes. In two of these patients (Pts 1 and 5), who showed significant activation in either IFN-γ spot count or activity by ELISpot and for whom plasma samples and plasma exosomes were available, we performed FACS and ELISpot analysis of DEX systemic transfer as shown in **Figure 5**.

### 3.7 Study of DEX translocation into the blood following DC vaccine

To investigate the possibility that plasma after neoantigen peptide-pulsed DC vaccine therapy contains vaccine-derived DEX, we analyzed the properties of exosomes in plasma after DC vaccine administration in the lymph nodes (**Figure 5**). Plasma was collected from patients 18–24 h after DC vaccine administration, and exosomes were extracted. The concentration of CD63+ exosomes (suspended in 200 μL phosphate-buffered saline [PBS]) isolated from 2 mL of plasma ranged from 5 to 30 ng/mL (**Figure 5A**; seven patient samples), and they were confirmed to be exosomes using western blotting for CD9 and CD63 (**Figure 5B**). To determine whether the plasma-derived exosomes were derived from DCs administered as vaccine, exosomes extracted from plasma collected and cryopreserved before and after vaccination (Pts. 1 and 5) were subjected to anti-CD9 antibody-bound microbeads and their surface antigen was analyzed by FACS. The expression levels of CD40, CD86, and HLA-DR on the plasma exosomes after administering the DC vaccine were higher than those in the pre-vaccine exosomes (**Figures 5C, D**). Furthermore, when exosomes were added to these patient PBMCs at a concentration of 1 ng/mL, IFN-γ ELISpot responses were increased in post-vaccine plasma-derived exosomes compared to pre-vaccine plasma exosomes (**Figure 5E**, data from Pt 1; **Figure 5F**, data from Pt 5).

## 4 Discussion

In this study, we analyzed the properties of DEX released from neoantigen peptide-pulsed DCs and reported that the addition of DEX *in vitro* transforms monocytes into antigen-presenting DC-like cells. Furthermore, we suggested the presence of DEX in patient plasma after neoantigen peptide-pulsed vaccine therapy, indicating that it may be involved in the amplification of immune responses in DC vaccine therapy. By analyzing the interaction of DEX extracted and cryopreserved from the culture supernatants of neoantigen-pulsed DCs from seven patients undergoing this therapy with the patient’s peripheral blood monocytes and lymphocytes, it was revealed that: ① DEX expresses the same surface markers as DCs and presents neoantigen. ② DEX is absorbed by autologous monocytes, which transform the monocytes in terms of surface antigen expression into DCs. ③DEX-engulfed monocytes present the same neoantigen as DCs and activate neoantigen-reactive T lymphocytes. These findings suggest that in intranodal neoantigen peptide-DC vaccine therapy, besides the direct activation of neoantigen-reactive T cells by neoantigen-pulsed DCs, DEX may also be incorporated into other antigen-presenting cells to activate neoantigen-reactive T cells.

Our findings are distinguished by several unique aspects. First, introducing exosomes from cultured DCs of patients undergoing neoantigen peptide-pulsed DC vaccine therapy into autologous resting monocytes modifies the monocytes’ surface antigens and enhances antigen presentation. Second, this effect occurs either through cross-dressing or the internalization of neoantigen-pulsed DC-derived DEX into monocytes, presenting neoantigen peptides. This presentation initiates the expression of cytokine and chemokine mRNAs and leads to the production of IL-12 and the activation of NF-κ B. Third, we observed that plasma exosomes in the initial period post neoantigen DC vaccine administration contained DC-derived exosomes, and these induced immune responses in PBMC, suggesting the potential for systemic transfer of DC-derived DEX.

There have been many reports on the release of DEX from antigen-presenting mature DCs, which is taken up by other antigen-presenting cells and activates antigen-specific T cells (24–30). However, these reports were based on mouse and *in vitro* experiments using an experimental system with Ovalbumin. Our study of DEX in *ex vivo* analysis of human clinical specimens showed that autologous monocytes took up DEX released from DCs and that these monocytes were transformed into DC-like antigen-presenting DCs.

There are two possible mechanisms for the increase in neoantigen-reactive T cells in the peripheral blood after neoantigen peptide-pulsed DC vaccine infusion therapy into the lymph node (LN). The first pathway involves DCs transferred into the LN, where they encounter lymphocytes already present or neoantigen-reactive naïve T lymphocytes that have migrated into the LN. This interaction results in lymphocyte proliferation in the lymph node, subsequent drainage through export lymph vessels or lymph node veins, and eventual circulation into the peripheral blood. The second pathway represents an indirect mechanism, wherein antigen-reactive T lymphocytes are activated by DEX released from DCs introduced into the lymph node. These DEX may be taken up by surrounding APSs in the LN or migrate to other tissues to be absorbed by other APCs.

Exosomes, including DEX, can migrate systemically and can be taken up by other APCs, such as monocytes, at sites other than the LNs (37, 38), which may activate antigen-presenting and antigen-reactive T lymphocytes and induce systemic amplification of the immune response.

When DEX binds to monocytes, whether it fuses to the monocyte surface (cross-dressing) or is incorporated into the monocyte and activates T cells is unknown; however, both pathways are probably involved. The mechanism by which major histocompatibility complex antigen complexes migrate to the cell surface is known as cross-dressing, and it has been reported that DEX induces cross-dressing in another DC (39). Similarly, DEX released from antigen-presenting DCs may present antigen to T cells via cross-dressing to antigen-presenting cells such as monocytes and macrophages (40, 41). Moreover, DC-associated surface antigens (CD86, CD40, and HLA-DR) are expressed on monocytes supplemented with DEX, indicating that DEX may cross-dress on monocytes. However, confocal microscopy observations suggest that some DEX was internalized into monocytes. Although the addition of neoantigen-pulsed DC-derived DEX to monocytes activated CD8+ lymphocytes in this study, DEX-pulsed monocytes loaded with a control peptide unrelated to the neoantigens did not have the same effect. Other similar studies have reported that subcutaneous administration of neoantigen-loaded serum exosomes to mice causes strong CD8 T lymphocyte responses *in vivo* and *in vitro* (42), and DC vaccines induce stronger immune responses compared to adjuvant vaccines, even with the same neoantigens (43). Considering that this experiment was performed when DC-derived DEX with Class-II affinity long peptide was added to monocytes, it is possible that DEX-pulsed monocytes specifically activate CD8 T lymphocytes that recognize the short epitope with Class-I affinity contained in the long peptide. The results of the experiment showing that DEX specifically activates CD 8+ lymphocytes that recognize the class-I affinity short epitope contained in the long peptide also support the possibility of DEX incorporation into monocytes (**Figure 4**). In addition, the results, which demonstrate an increase in T-cell activation in a time-dependent and dose-dependent manner upon addition DEX to monocytes (**Supplementary Figures 3A, B**), suggest that a portion of DEX is incorporated into the activated monocytes. Cross presentation, the ability to intracellularly reprocess antigens captured by phagocytosis and present them to CD8+ T lymphocytes on Class I cells, which is a major functional feature of DCs (44), supports the possibility that DEX induces monocytes to become dendritic-like cells. Although human peripheral blood monocytes have poor cross-presentation ability, macrophages in ascites fluid have recently been reported to cross-present, and it has been shown that monocyte-derived DCs stimulated by cytokines acquire cross-presentation ability (45). These findings suggest that monocytes with low cross-presentation ability are transformed into DC-like cells by the uptake of DEX. Further studies are needed.

The details of how DEX, when internalized into monocytes, induces monocytes to functionally resemble antigen-presenting mature DCs are unknown. It has been reported that NF-kB activation and IL-12 production are involved in DC maturation (46–48). In the present study, preliminary results show that the DEX uptake by monocytes induces NF-kB activation (nuclear translocation of P65) and increases intracellular IL-12 (**Supplementary Figure 4**). In addition, the results of gene expression studies of monocytes induced by the addition of DEX also showed an increase in gene expression of various chemokines and chemokine receptors (**Supplementary Table 1**). Moreover, it has been reported that the expression of various chemokine receptors increases with the maturation of DCs (49, 50). Taken together, these findings suggest that DEX uptake into monocytes induces intracellular signaling and protein production, similar to the maturation process of DCs. However, the experimental data are preliminary and require further detailed analysis.

It remains unclear whether the DEX released within the lymph node acts locally or disperses to exert systemic effects. When DC vaccines are administered into the lymph node, as in our method, DEX released from these cells is likely absorbed by other APCs within the lymph node, thereby activating T cells. Evidence supporting the potential migration of DEX into plasma includes: 1) when pre- and post-vaccine peripheral blood monocytes were co-cultured with lymphocytes, post-vaccine monocytes tended to stimulate lymphocytes more strongly; 2) elevated levels of HLA-DR, CD40, and CD86 molecules in plasma-derived exosomes within 24 h post-vaccination.

Additionally, plasma exosomes introduced to post-vaccination PBMCs increased IFN-γ production (**Figure 5**). Although our data indicate that post-vaccination plasma may contain DEX (**Figure 5**), this does not provide direct evidence of DEX migration from lymph nodes to other body parts and initiating an immune response. Although it has been reported that human plasma contains fewer HLA-containing exosomes (51), our analysis showed that DEX expressing HLA-DR, usually carried by antigen-presenting cells, increased after vaccination. In the mouse model, DEX migrates to LNs and is released into the microenvironment. Moreover, it has been reported that DEX influences the surrounding APCs (52) by this mechanism and that exosomes derived from DCs are absorbed by other DCs, transferring antigens and miRNA to those cells (53).

In addition, it has been reported that porcine milk exosomes are incorporated into monocytes by phagocytosis (54). Furthermore, Obregon et al. reported that in humans, exosomes released from matured DCs derived from monocytes of healthy volunteers fuse to resting DCs, which can present as alloantigens (55). These reports, in general, support the possibility that DEX may be involved in systemic immune responses through migration. Our results indicate that DEX may be present in plasma at 24 h after vaccine administration in DC vaccine therapy. However, the temporal and spatial distribution of DEX remains unknown, and further analysis is needed to prove the presence of antigenic peptides by proteomic analysis of plasma exosomes and other methods. More detailed analysis is needed in the future, such as demonstrating the presence of antigen peptides by proteomic analysis of plasma exosomes. Moreover, considering the reported immunosuppressive effect of DEX released from IL-10-stimulated DCs (56), it is essential to investigate the immunostimulatory and immunosuppressive effects of DEX in cancer vaccine therapy in future studies.

Analysis of samples from patients receiving neoantigen peptide-pulsed DC vaccine therapy indicates that DEX may play a role in immune amplification. However, the relationship between the immune amplifying effect of DEX and the efficacy of this therapy is unknown. DC-derived exosomes pulsed with tumor antigens were reported to inhibit tumor growth in a T cell-dependent manner in a mouse model more than 20 years ago (31). Based on those results, attempts to use DEX as a cancer vaccine were initiated, and treatment of non-small cell lung cancer and melanoma with DEX released from monocyte-derived DCs was evaluated. However, the efficacy was reported to be limited (32–34). In future studies, we intend to use DEX as a Bio-Marker to predict efficacy rather than as a therapeutic tool such as a vaccine.

No studies have examined the involvement of plasma DEX in the efficacy of the DC vaccine. Muller et al. reported longer survival in patients with glioma who had higher cytokine mRNA levels in plasma exosomes after peptide-pulsed DC vaccine. However, it was not clear whether the exosomes were DEX (57). In our study, we did not demonstrate direct evidence that the plasma exosomes originated from DCs; however, the presence of exosomes expressing HLA-DR and CD86 indicates the potential for some to contain DEX. The correlation between DEX mRNA expression and prognosis warrants additional investigation. The DEX analyzed in this research was derived from DC culture supernatants of patients undergoing neoantigen peptide-pulsed DC vaccine therapy. Further detailed analysis is required to ascertain whether the *in vitro* and *ex vivo* immunological effects of DEX correspond with clinical outcomes. Additionally, future studies are necessary to evaluate the immunological impact of DEX in patients and to determine if the quality of DEX influences its clinical effectiveness.

The purpose of this study was to analyze the immunological role of DEX through a detailed analysis of immune cells and DEX in patients receiving neoantigen peptide-pulsed DC vaccine infusion therapy into lymph nodes. However, several limitations affect the evaluation and significance of the study’s results. First, the results demonstrate the potential of DEX as a neoantigen carrier but do not establish its utility as a vaccine. Furthermore, as this study did not show direct evidence of DEX transfer to the whole body, a detailed analysis of DEX disposition in the body following LN administration of the DC vaccine is necessary but challenging to conduct in humans. Additionally, this study involves a small cohort of patients who received neoantigen-pulsed DC vaccines alongside standard therapies and chemotherapy and exhibited clinical responses. Thus, the limited number of samples and the availability of sufficient samples for analysis, coupled with the need for consent for neoantigen analysis and immunological response assessments, constrain the study. Consequently, further research using animal models is essential.

## 5 Conclusion

This study suggests that (1) DC-derived exosomes presenting neoantigen peptides, which are currently the focus of much attention, migrate to autologous monocytes and transform into DC-like cells that can present these peptides, and (2) DEX may play a role in amplifying immune responses to tumor antigens in neoantigen DC vaccine therapy, which has been reported to be safe and immunologically effective. Looking ahead, the effective utilization of DC culture supernatants should be considered in clinical and research settings, with further investigation warranted with more cases.

## Data Availability

The datasets presented in this article are not readily available because the genetic data present in the article contains personal information and therefore cannot be registered in a public repository. Requests to access the datasets should be directed to the corresponding author.
