# Supplementary material for: Dendritic cell-derived exosomes induce monocyte antigen-presentation and immune amplification in neoantigen vaccine therapy
Source: Front Immunol. 2025 May 19;16:1565696. doi: 10.3389/fimmu.2025.1565696 (PMC12127191; doi:10.3389/fimmu.2025.1565696)
Supplement: Supplementary file 1 [file Table1.docx]

Supplementary Material

Dendritic cell-derived exosomes induce monocyte antigen-presentation and immune amplification in neoantigen vaccine therapy

**Shinji Morisaki1,2, Hideya Onishi3*, Takafumi Morisaki3, Makoto Kubo3, Masayo Umebayashi1, Hiroto Tanaka1, Norihiro Koya1, Shinichiro Nakagawa1, Kenta Tsujimura1, Sachiko Yoshimura4, Poh Yin Yew4, Kazuma Kiyotani5, Yusuke Nakamura5, Masafumi Nakamura3, Takehiro Torisu2, Takanari Kitazono2, and Takashi Morisaki1**

*** Correspondence:** Hideya Onishi: ohnishi.hideya.928@m.kyushu-u.ac.jp

## Supplementary Figures


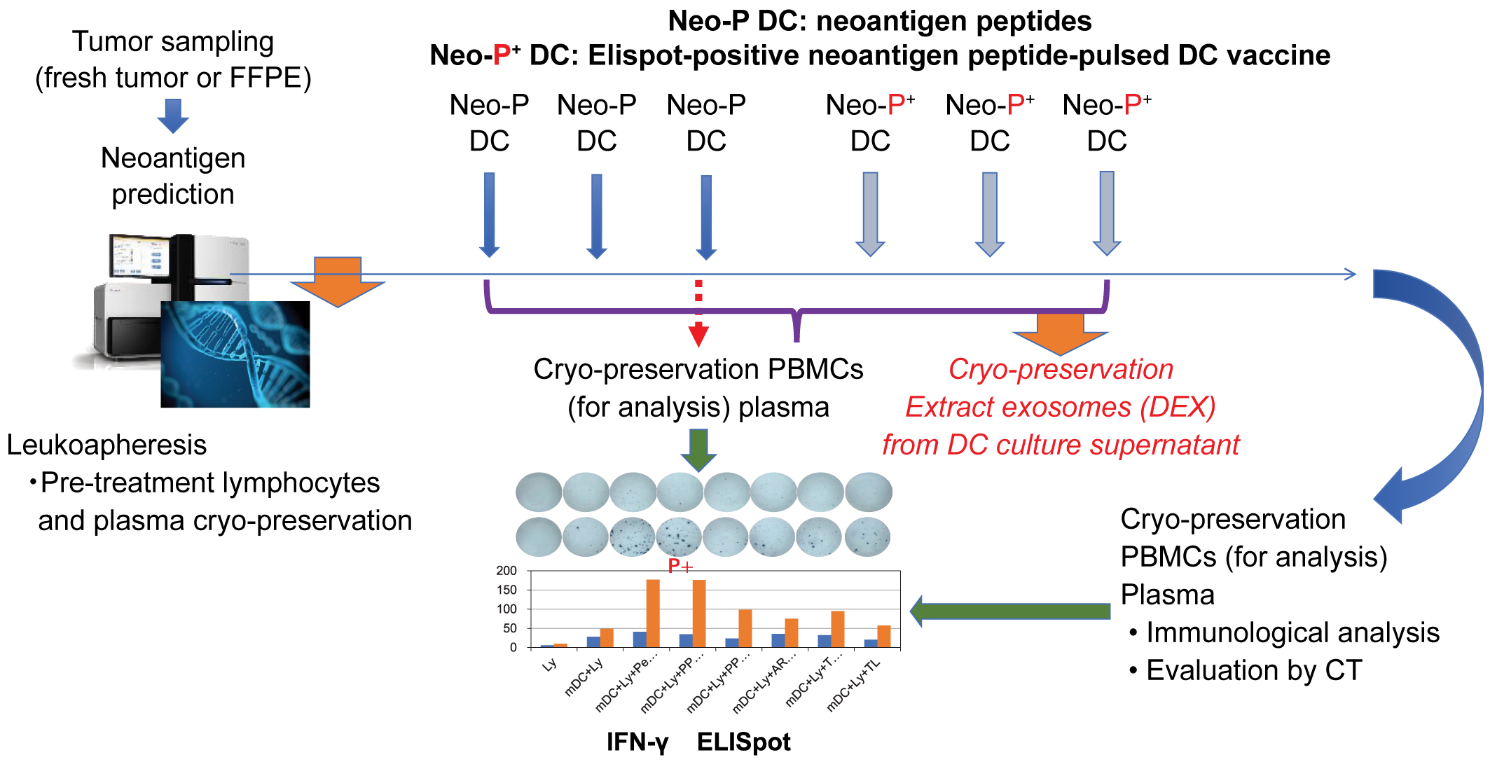


**Supplementary Fig. 1** **Protocol for intranodal DC therapy and DEX collection and preservation.** Neoantigen analysis will be conducted on the patient's tumor specimen in advance. Lymphocytes will also be collected prior to treatment through apheresis. The vaccine therapy will involve administering pulsed neoantigen to monocyte-derived dendritic cells three times initially, followed by another three doses with an increased amount of the peptide, to verify which neoantigen peptide induces T cell response by ELISpot technique. Supernatants from the dendritic cell cultures collected during vaccine preparation were cryopreserved. Exosomes in these supernatants were later extracted using a stepwise ultracentrifugation method.

**
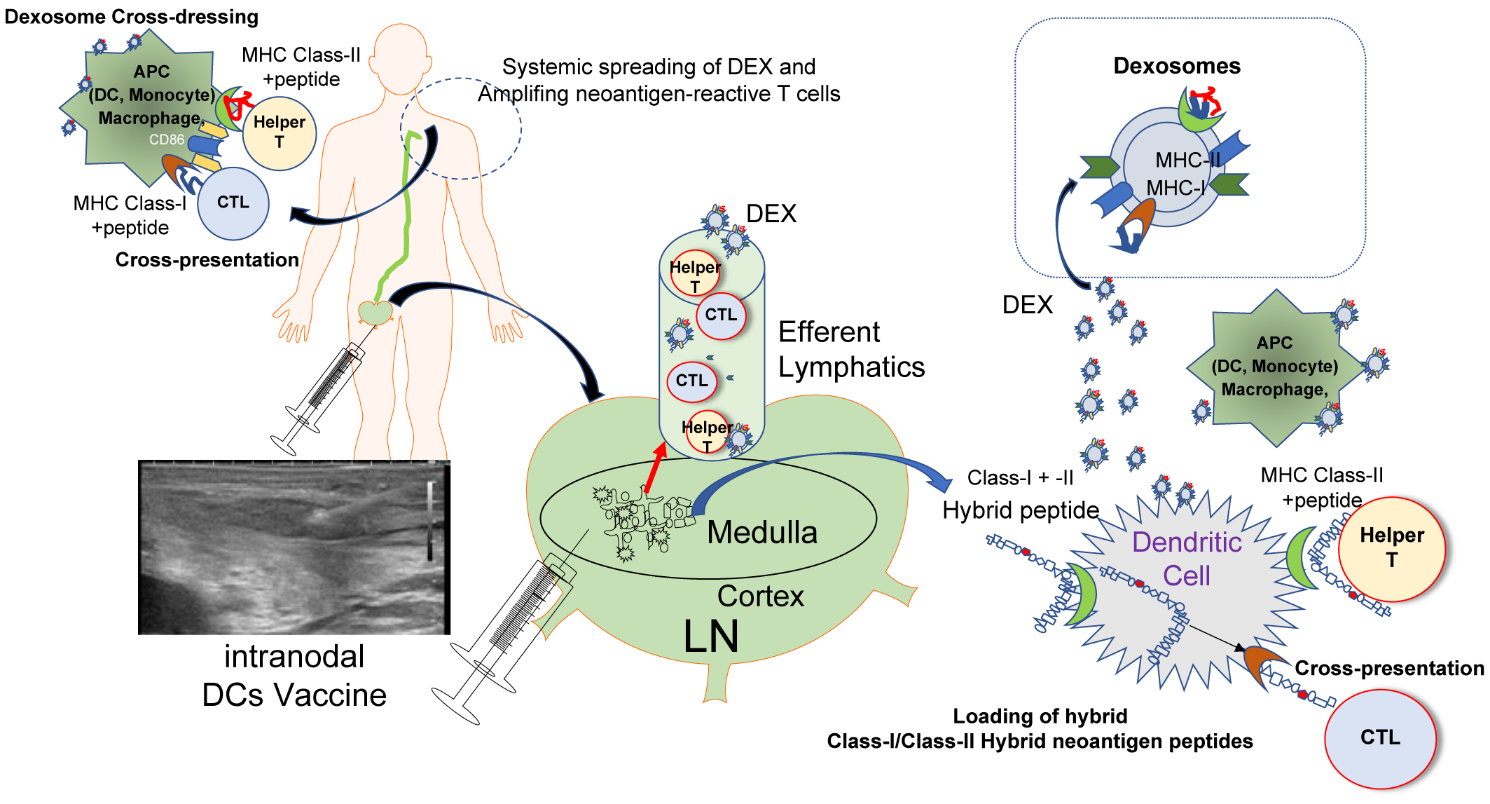
**

**Supplementary Fig. 2 Mechanism of systemic spread of DEX and activation of neoantigen-responsive lymphocytes by intralymphatic administration of neoantigen-pulsed dendritic cells (schematic diagram, overall view of this study)**

**
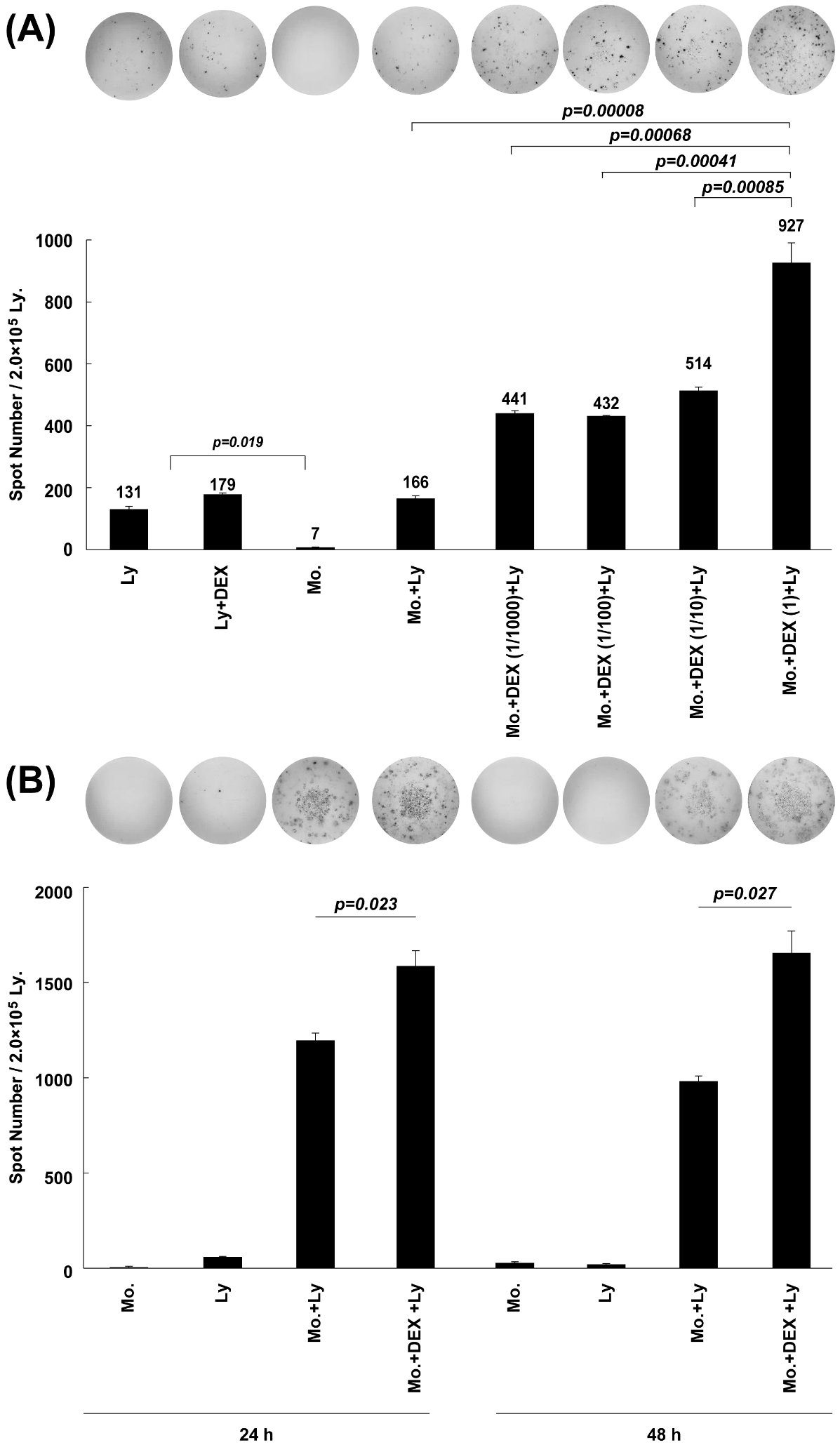
**

**Supplementary Fig. 3** **Dose- and time-dependent activation of antigen-responsive T lymphocytes upon addition of DEX to monocytes.** (A) DEX induces monocyte-mediated presentation of neoantigens to T lymphocytes (isolated from patient 1) in a concentration-dependent manner. T lymphocytes were isolated from PBMCs collected and cryopreserved after 0 vaccination and added to monocytes pulsed with peptide (+)DEX and cultured for 48 h to obtain ELISpot responses. Controls were T lymphocytes alone, T lymphocytes + DEX, monocytes alone, T lymphocytes + monocytes, and DEX diluted to 1/1,000th, 1/100th, or 1/10th of the original concentration was added to T lymphocytes + monocytes to compare groups. The results showed that DEX stimulated the activation of neoantigen-responsive T lymphocytes via monocytes in a concentration-dependent manner. (B) Similarly, T lymphocytes were isolated and added to monocytes pulsed with peptide (+)DEX and cultured for 24 and 48 h to obtain ELISpot responses. Control groups were T lymphocytes alone, monocytes alone, and T lymphocytes + monocytes. The results showed that DEX stimulated the activation of neoantigen-responsive T lymphocytes via monocytes after 24 h, and the effect continued at least until 48 h later.

**
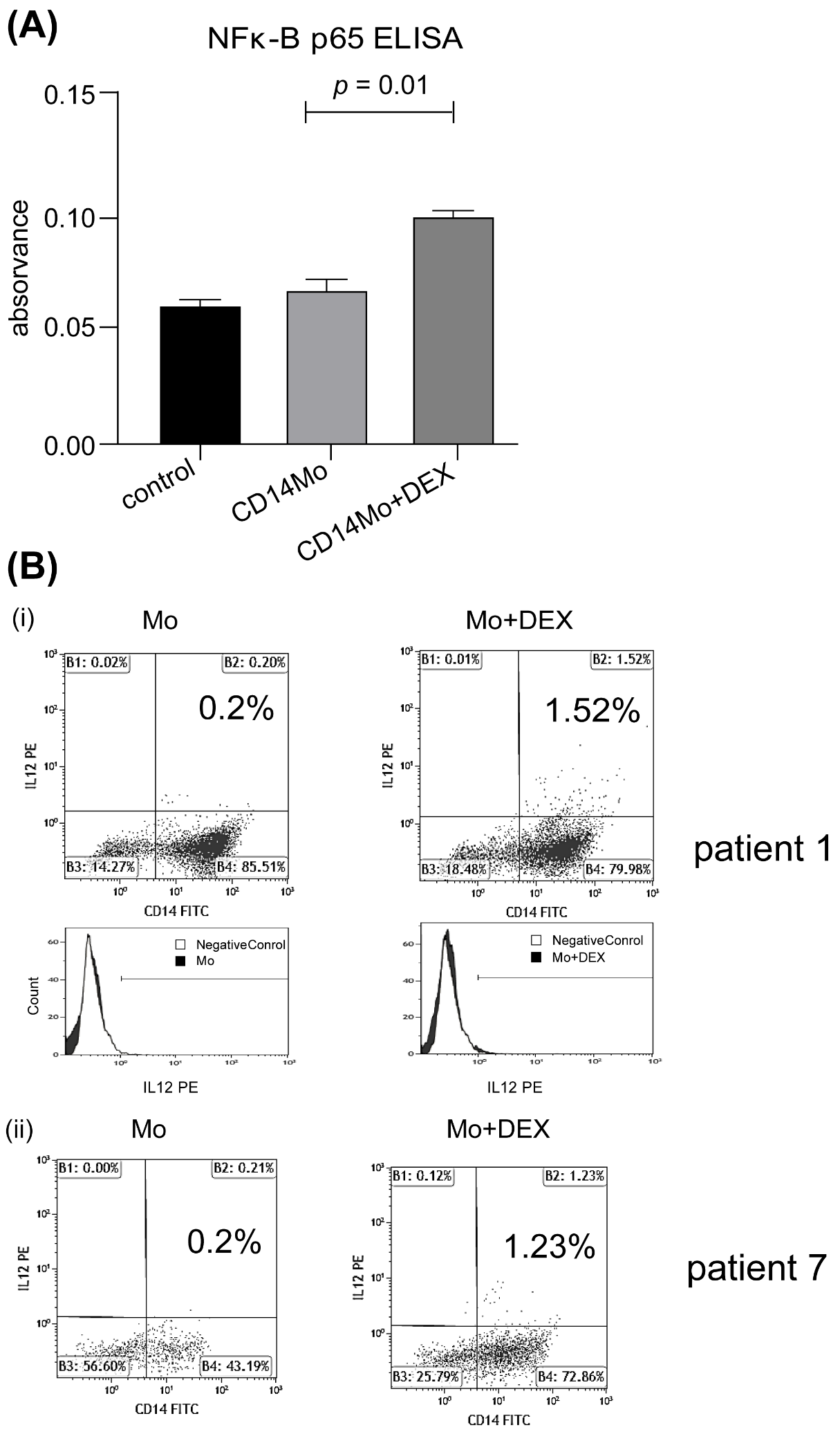
**

**Supplementary Fig. 4** **DEX-induced enhancement of NFκB p65 activation and IL-12 production in CD14 monocytes.** As shown in (A), the absorbance of NFκβ p65 in monocytes treated with peptide-DEX was significantly increased compared to controls. These results indicate that exosomes released from DCs pulsed with neoantigen peptide activate p65 by nuclear translocation of NFκβ in CD14-positive Mo. As shown in (B), there was an increase in the percentage of IL-12 positivity within monocytes supplemented with peptide-DEX compared to controls (Pt. 1: DEX group; 1.52%, control; 0.20, Pt. 7: DEX group; 1.23%, control; 0.21). The results indicate that exosomes released from DCs pulsed with neoantigen peptides enhance IL-12 production within CD14-positive Mo.


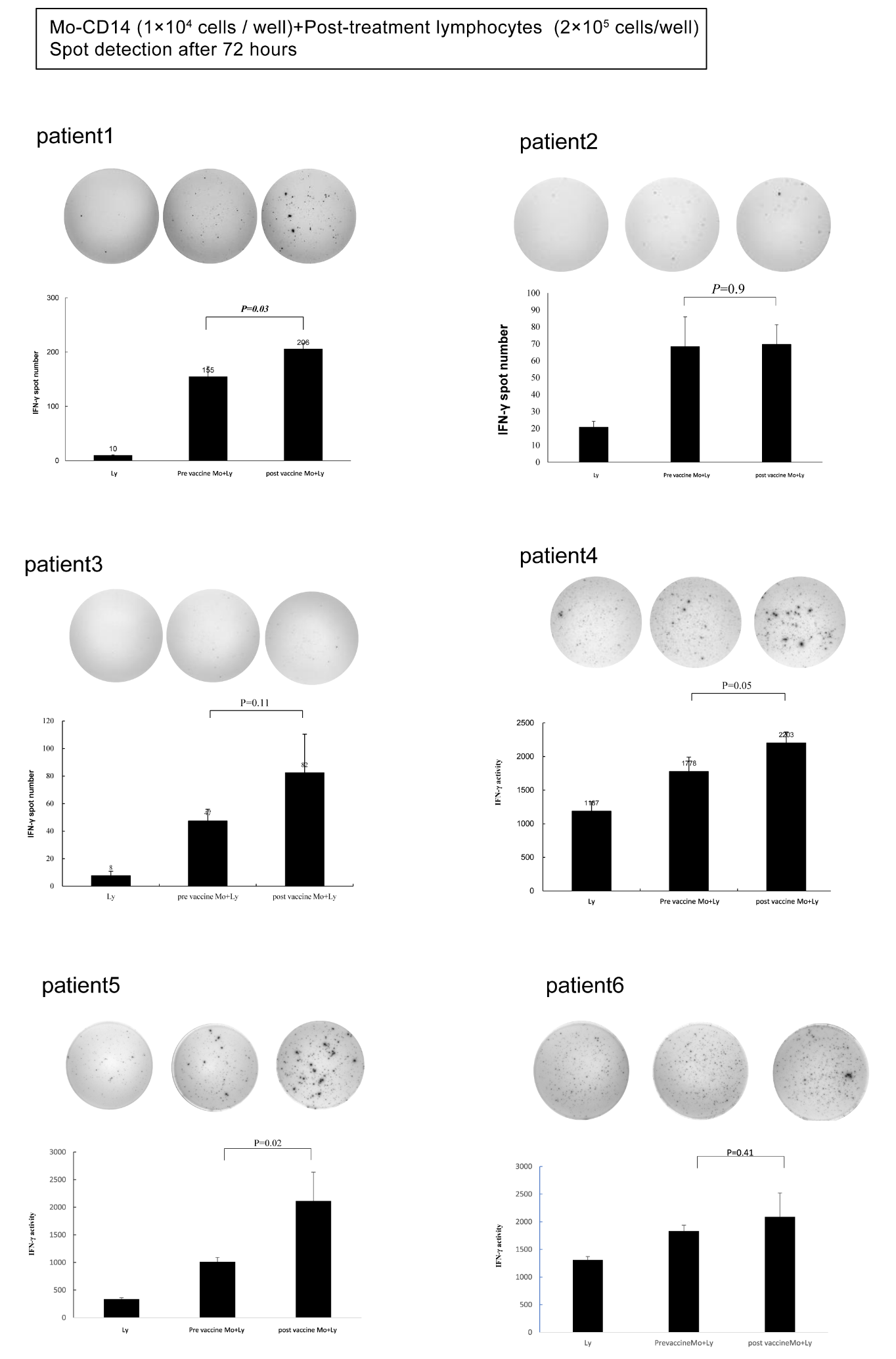


**Supplementary Fig. 5 ELISpot response of CD14+ monocytes before and after vaccine plus post-vaccine lymphocytes. IFN**-γ spot counts and activity results for each patient are shown, as well as CD14+ monocyte and lymphocyte counts and reaction times in the figure.


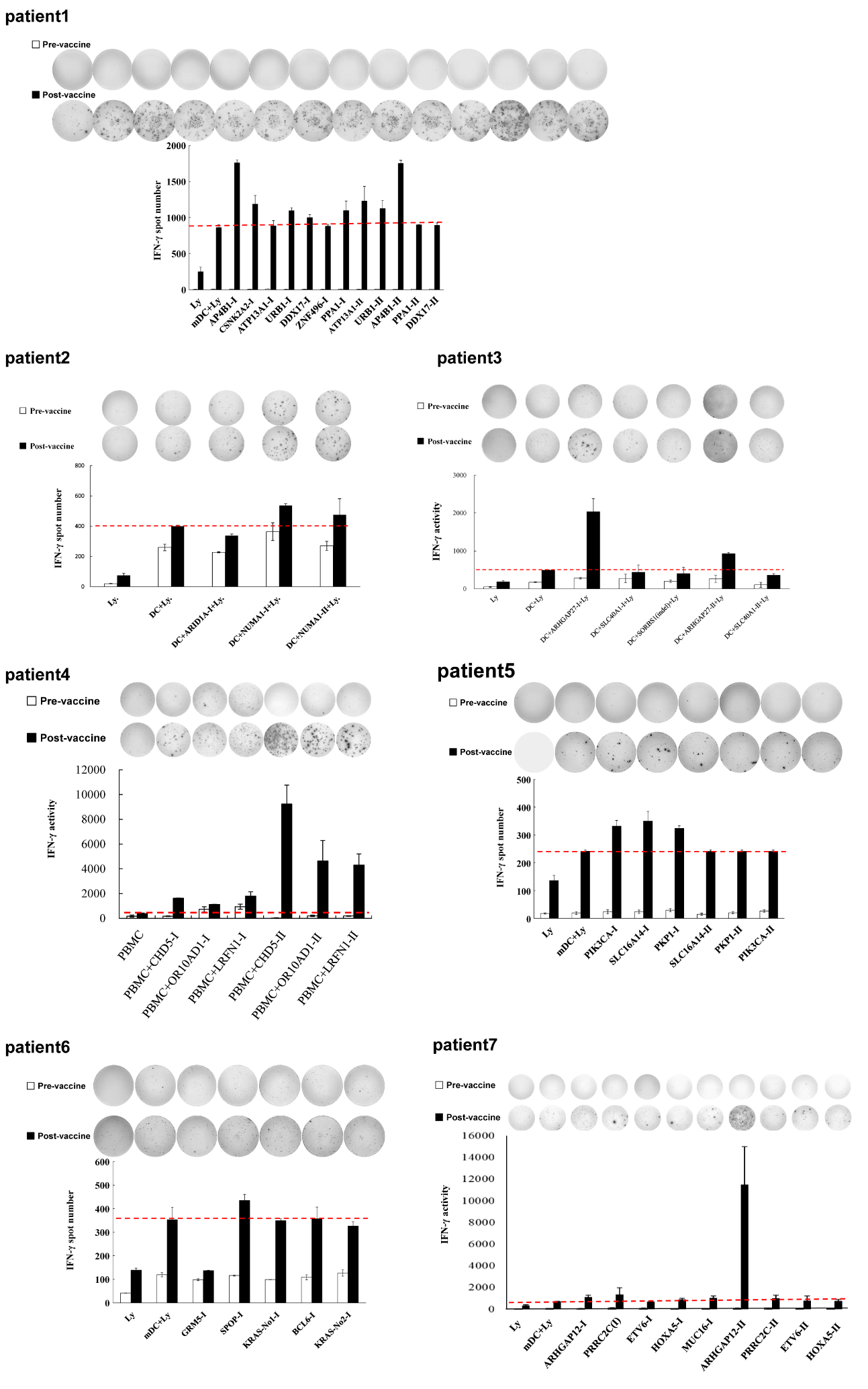


**Supplementary Fig. 6 Pre- and post-vaccine lymphocyte responses to each neoantigen in seven patients (ELISPOT).** The top of each spot diagram shows before treatment and the bottom shows after treatment. The number of IFN-γ spots or activity was evaluated and a bar above the red dotted baseline (no peptide added) was defined as an immune response. Neoantigen was added to PBMCs before and after treatment only in patient 4. In all other cases, mDC (mature dendritic cells) and neoantigen were added to the Ly(lymphocytes) before and after treatment. Hyphenated 'I' after each gene name indicates short neoantigen peptide and 'II 'indicates long peptide.


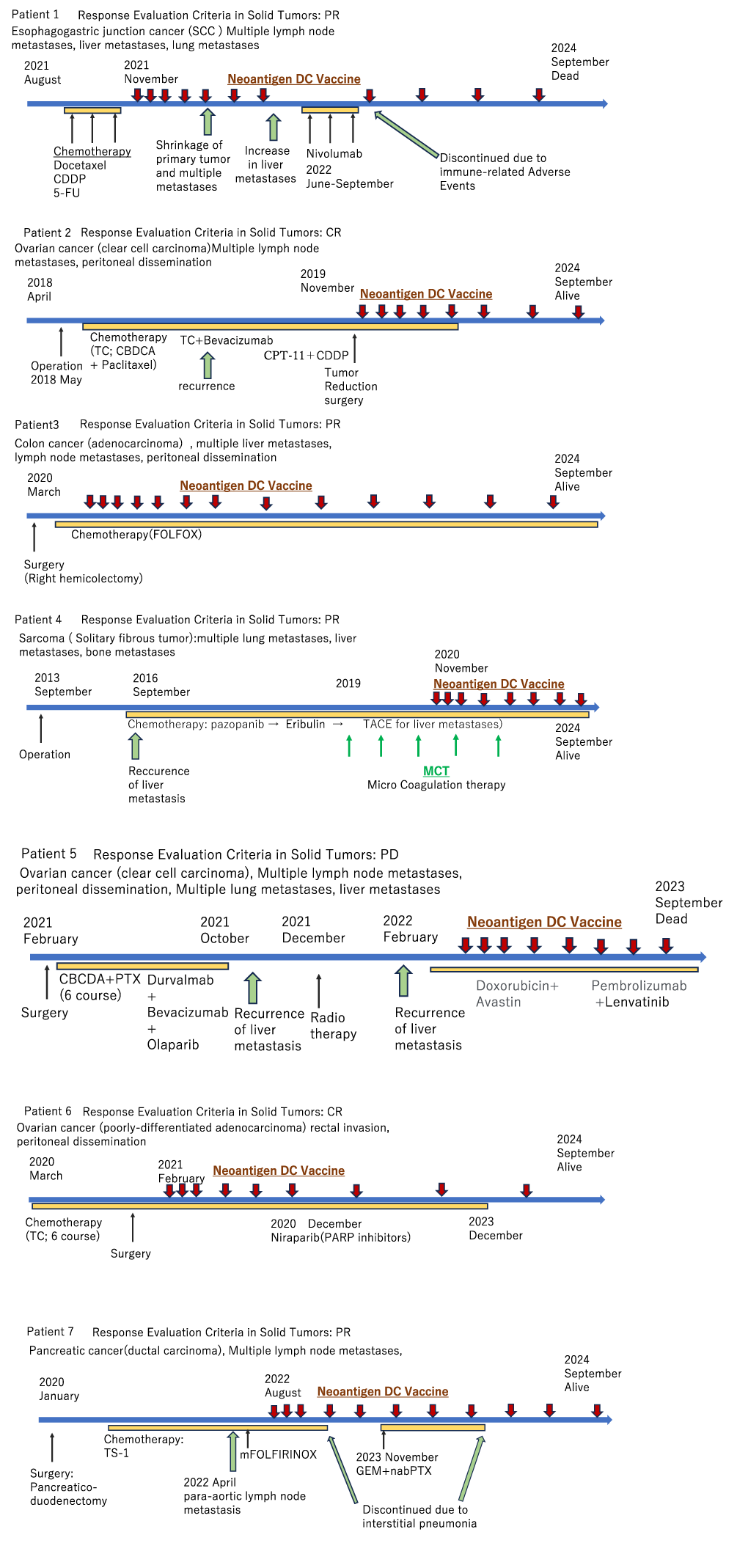


**Supplementary Fig. 7** **The simplified treatment process for each patient is illustrated in the flowchart.** Patien**t** number, disease name, metastasis, and treatment efficacy (initial response) are listed at the top. Brown arrows indicate the period of neoantigen vaccine and yellow bars indicate the period of chemotherapy. Other arrows across the bars indicate the timing of surgery and disease assessment events.

**
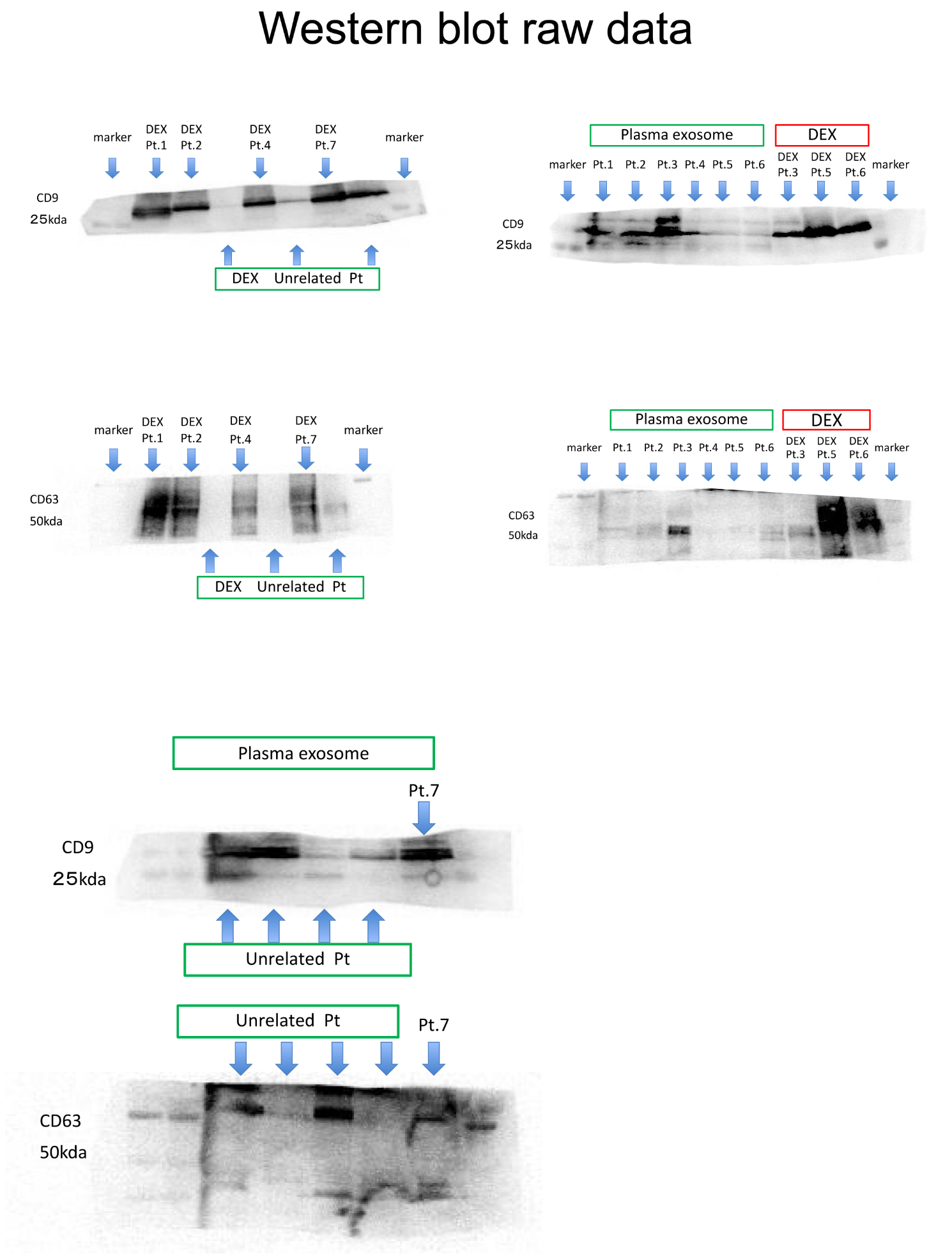
**

**Supplementary Fig. 8**. **Western blot diagram of each patient (pt). Sample figures for patients unrelated to the current study (unrelated pt) are also included. Each blot is indicated by an arrow with the patient number.**

1. **Supplementary Tables**

**Supplementary Table 1.** Increased expression of immune-related genes by DEX incorporation in monocytes (transcripts per million)kl.

| **Oncoantigen genes** | | |
| --- | --- | --- |
| Gene | Mo | Mo+DEX |
| CDC45L | 0.07 | 0.10 |
| CDCA1 | 0.36 | 0.06 |
| CDH3 | 0.00 | 0.00 |
| DEPDC1 | 0.02 | 0.01 |
| ECT2 | 1.89 | 1.35 |
| FOXmM1 | 0.38 | 0.09 |
| GPC3 | 0.54 | 0.48 |
| HIG2 | 2.98 | 3.71 |
| HJURP | 0.00 | 0.02 |
| KIF20A | 0.00 | 0.04 |
| KNTC2 | 0.27 | 0.48 |
| KOC1 | 0.30 | 0.14 |
| MELK | 0.28 | 0.17 |
| MPHOSPH1 | 1.08 | 0.75 |
| NEIL3 | 0.04 | 0.03 |
| RNF43 | 0.09 | 0.01 |
| SPARC | 8.31 | 14.51 |
| TOMM34 | 15.60 | 21.41 |
| TOPK | 0.00 | 0.05 |
| UBE2T | 0.52 | 0.60 |
| URLC10 | 1.21 | 1.05 |
| WDRPUH | 0.00 | 0.00 |
| **Genes related to antigen presentation to T cells** | | |

| B2M | 24659.59 | 21526.16 |
| --- | --- | --- |
| CALR | 599.79 | 654.67 |
| CANX | 290.43 | 312.23 |
| HLA-A | 3265.14 | 3193.79 |
| HLA-B | 12991.11 | 14276.91 |
| HLA-C | 1029.19 | 1078.53 |
| HLA-DPA1 | 118.36 | 73.59 |
| HLA-DPB1 | 250.44 | 163.21 |
| HLA-DQA1 | 26.20 | 19.62 |
| HLA-DQA2 | 23.46 | 16.31 |
| HLA-DQB1 | 17.42 | 14.47 |
| HLA-DRA | 1102.90 | 765.85 |
| HLA-DRB1 | 211.18 | 161.69 |
| HLA-DRB5 | 15.34 | 10.80 |
| HLA-E | 1541.56 | 1638.99 |
| HLA-F | 506.36 | 476.38 |
| HLA-G | 0.18 | 0.34 |
| HLA-H | 3.54 | 10.12 |
| IFNA1 | 0.00 | 0.00 |
| IFNA2 | 0.00 | 0.00 |
| IFNB1 | 0.05 | 0.00 |
| IFNG | 0.44 | 0.47 |
| IRF1 | 100.24 | 96.22 |
| MICA | 21.22 | 23.30 |
| MICB | 17.78 | 17.07 |
| PSMB8 | 181.17 | 202.68 |
| PSMB9 | 110.13 | 112.97 |
| TAP1 | 155.62 | 183.74 |
| TAP2 | 42.18 | 39.07 |
| TAPBP | 119.64 | 111.38 |
| **Genes associated with immune cells (T cells, B cells, antigen-presenting cells)** | | |

| CD19 | 0.85 | 0.69 |
| --- | --- | --- |
| CD28 | 3.40 | 2.88 |
| CD4 | 36.95 | 11.95 |
| CD68 | 1615.53 | 1655.40 |
| CD80 | 1.21 | 2.71 |
| CD86 | 134.81 | 141.40 |
| CD8A | 29.23 | 35.24 |
| CD8B | 20.01 | 20.16 |
| IL2RA | 0.60 | 1.09 |
| **Genes associated with antigen recognition and activation of T cells** | | |
| CD247 | 34.51 | 35.35 |
| CD27 | 8.10 | 8.23 |
| CD3D | 27.59 | 36.39 |
| CD3E | 48.26 | 53.81 |
| CD3G | 6.86 | 8.34 |
| GZMA | 33.81 | 30.46 |
| GZMB | 52.72 | 62.01 |
| ICOS | 0.63 | 0.83 |
| LAMP1 | 290.28 | 417.21 |
| TNFRSF9 | 6.78 | 3.87 |
| **Chemokines/cytokines gene** | | |
| CCL17 | 0.09 | 0.19 |
| CCL19 | 0.21 | 0.00 |
| CCL2 | 61.79 | 95.05 |
| CCL20 | 6.03 | 9.07 |
| CCL21 | 0.00 | 0.00 |
| CCL22 | 10.09 | 19.00 |
| CCL4 | 184.02 | 241.28 |
| CCL5 | 359.17 | 413.77 |
| CCR10 | 0.08 | 0.13 |
| CCR4 | 3.58 | 3.99 |
| CCR5 | 169.59 | 269.92 |
| CCR6 | 0.38 | 0.30 |
| CCRL2 | 133.70 | 211.57 |
| CD40 | 16.89 | 23.68 |
| CD40LG | 1.03 | 1.19 |
| CD70 | 0.71 | 0.95 |
| CXCL10 | 0.81 | 2.03 |
| CXCL11 | 0.07 | 0.08 |
| CXCL13 | 0.98 | 1.33 |
| CXCL9 | 0.15 | 0.13 |
| FASLG | 2.25 | 3.36 |
| IL10 | 0.56 | 0.26 |
| IL12A | 0.13 | 0.22 |
| IL12RB2 | 0.45 | 0.48 |
| IL17A | 0.00 | 0.00 |
| IL17RB | 0.05 | 0.04 |
| IL23A | 2.38 | 2.59 |
| IL6 | 2.15 | 3.13 |
| PRF1 | 56.98 | 56.51 |
| TBX21 | 4.81 | 5.29 |
| TGFB2 | 0.14 | 0.15 |
| TGFB3 | 0.19 | 0.13 |
| TNF | 37.55 | 64.40 |
| TNFSF1 | 1.18 | 0.94 |
| TNFSF10 | 7.82 | 11.73 |
| TNFSF11 | 0.11 | 0.36 |
| TNFSF12 | 13.59 | 9.62 |
| TNFSF13 | 18.70 | 17.69 |
| TNFSF13B | 21.50 | 25.66 |
| TNFSF14 | 15.17 | 51.98 |
| TNFSF15 | 10.43 | 21.97 |
| TNFSF18 | 0.80 | 0.84 |
| TNFSF3 | 72.84 | 70.56 |
| TNFSF4 | 0.56 | 0.87 |
| TNFSF8 | 34.43 | 36.02 |
| TNFSF9 | 1.93 | 2.13 |
| **Cytokine receptor** | | |

| TNFRSF10A | 20.02 | 21.28 |
| --- | --- | --- |
| TNFRSF10B | 48.44 | 36.70 |
| TNFRSF10C | 2.30 | 2.16 |
| TNFRSF10D | 12.13 | 13.00 |
| TNFRSF11A | 1.88 | 1.23 |
| TNFRSF11B | 0.00 | 0.04 |
| TNFRSF12A | 21.58 | 32.79 |
| TNFRSF13B | 0.33 | 0.37 |
| TNFRSF13C | 0.34 | 0.28 |
| TNFRSF14 | 84.36 | 75.29 |
| TNFRSF16 | 0.35 | 0.45 |
| TNFRSF17 | 0.37 | 0.24 |
| TNFRSF19 | 0.02 | 0.02 |
| TNFRSF19L | 27.60 | 30.58 |
| TNFRSF1A | 205.22 | 178.04 |
| TNFRSF1B | 357.98 | 362.12 |
| TNFRSF21 | 4.97 | 8.36 |
| TNFRSF25 | 1.77 | 1.71 |
| TNFRSF27 | 0.08 | 0.03 |
| TNFRSF3 | 82.60 | 90.99 |
| TNFRSF6 | 13.27 | 11.18 |
| TNFRSF6B | 2.34 | 3.53 |
| TNFRSF8 | 2.89 | 1.89 |
| **Genes related to immunosuppressor cells** | | |

| CTLA4 | 1.58 | | 1.05 | |
| --- | --- | --- | --- | --- |
| FOXP3 | 0.62 | | 0.96 | |
| TGFB1 | 270.64 | | 471.38 | |
| **Immune checkpoint molecule genes** | | | | |
| BTLA | | 0.74 | | 0.54 |
| C10orf54 | | 238.04 | | 122.79 |
| PD-L1 (CD274) | | 15.00 | | 12.37 |
| CD276 | | 50.02 | | 84.84 |
| HAVCR2 | | 78.95 | | 72.44 |
| IDO1 | | 11.12 | | 12.70 |
| LAG3 | | 1.90 | | 2.37 |
| PD1 (PDCD1) | | 1.01 | | 1.53 |
| PD-L2 (PDCD1LG2) | | 1.24 | | 0.56 |
| TIGIT | | 6.87 | | 6.70 |

**Supplementary Table 2.** Composition table of neoantigen peptides used for vaccine therapy in each patient. This table includes information on th**e** gene corresponding to each neoantigen, mutated amino acid and its position, HLA type, and mRNA expression level.

| patient1 |  |  |  |  |  |  |  |  |  |  |  |  |
| --- | --- | --- | --- | --- | --- | --- | --- | --- | --- | --- | --- | --- |
| peptide No. | gene | amino_acid | length | pos | peptide_mut | affinity_mut(nM) | peptide_wt | affinity_wt(nM) | HLA | tumor_var(rna) | tumor_exome(ref,var,freq) | normal_exome(ref,var,freq) |
| 1 | AP4B1-Ⅰ | Y75C | 10 | 7 | YLYMCTCAPL | 13 | YLYMCTYAPL | 13 | HLA-C03:04 | 20 | 47,22,0.319 | 50,0,0.000 |
| 2 | CSNK2A2-Ⅰ | K103N | 10 | 7 | KLIDTVNDPV | 8 | KLIDTVKDPV | 16 | HLA-A02:01 | 40 | 81,21,0.206 | 87,0,0.000 |
| 3 | ATP13A1-Ⅰ | F268L | 9 | 3 | SVLTLSMLV | 31 | SVFTLSMLV | 23 | HLA-A02:01 | 13 | 133,45,0.253 | 249,0,0.000 |
| 4 | URB1-Ⅰ | L834F | 9 | 6 | ALCLLFQAY | 45 | ALCLLLQAY | 107 | HLA-B15:01 | 16 | 138,48,0.258 | 178,0,0.000 |
| 5 | DDX17-Ⅰ | A652S | 11 | 3 | YTSQEYGAGTY | 47 | YTAQEYGAGTY | 40 | HLA-B15:01 | 258 | 104,60,0.366 | 122,0,0.000 |
| 6 | ZNF496-Ⅰ | F249L | 9 | 1 | LIIGEDYGV | 50 | FIIGEDYGV | 6 | HLA-A02:01 | 12 | 33,18,0.353 | 69,0,0.000 |
| 7 | PPA1-Ⅰ | D165V | 11 | 11 | KVIAINVDDPV | 144 | KVIAINVDDPD | 19733 | HLA-A02:01 | 442 | 28,9,0.243 | 19,0,0.000 |
| 8 | ATP13A1-Ⅱ | F268L | 18 | 5 | YYSVLTLSMLVAFEASLV | 6.4 | YYSVFTLSMLVAFEASLV | 6.2 | DRB1:0901 | 13 | 133,45,0.253 | 249,0,0.000 |
| 9 | URB1-Ⅱ | L834F | 15 | 8 | PLALCLLFQAYDKLE | 22.49 | PLALCLLLQAYDKLE | 16.48 | DRB1:1202 | 16 | 138,48,0.258 | 178,0,0.000 |
| 10 | AP4B1-Ⅱ | Y75C | 18 | 12 | QKKLVYLYMCTCAPLKPD | 24 | QKKLVYLYMCTYAPLKPD | 22 | DRB1:0901 | 20 | 47,22,0.319 | 50,0,0.000 |
| 11 | PPA1-Ⅱ | D165V | 18 | 14 | TDWKVIAINVDDPVAANY | 243.2 | TDWKVIAINVDDPDAANY | 3326.9 | DRB1:0901 | 442 | 28,9,0.243 | 19,0,0.000 |
| 12 | DDX17-Ⅱ | A652S | 18 | 12 | GAAAYGTSSYTSQEYGAG | 364.5 | GAAAYGTSSYTAQEYGAG | 230.6 | DRB1:0901 | 258 | 104,60,0.366 | 122,0,0.000 |
|  |  |  |  |  |  |  |  |  |  |  |  |  |
| **patient2** |  |  |  |  |  |  |  |  |  |  |  |  |
| peptide No. | gene | amino_acid | length | pos | peptide_mut | affinity_mut(nM) | peptide_wt | affinity_wt(nM) | HLA | tumor_var(rna) | tumor_exome(ref,var,freq) | normal_exome(ref,var,freq) |
| 1 | ARID1A-Ⅰ | H2248fs | 9 | * | ELIGFTSHL | 78 | * | * | HLA-A26:03 | 50 | 178,44,0.198 | 174,0,00.000 |
| 2 | NUMA1-Ⅰ | R1008W | 11 | 4 | EVAWLTQERGR | 297 | EVARLTQERGR | 311 | HLA-A26:03 | 29 | 180,30,0.143 | 139,0,0.000 |
| 3 | NUMA1-Ⅱ | R1008W | 18 | 7 | QEREVAWLTQERGRAQAD | 415 | QEREVARLTQERGRAQAD | 980.2 | DRB1:0901 | 29 | 180,30,0.143 | 139,0,0.000 |
|  |  |  |  |  |  |  |  |  |  |  |  |  |
| **patient3** |  |  |  |  |  |  |  |  |  |  |  |  |
| No. | gene | amino_acid | length | pos | peptide_mut | affinity_mut(nM) | peptide_wt | affinity_wt(nM) | HLA | mRNA_expression_data_from_TCGA(median) | tumor_exome(ref,var,freq) | normal_exome(ref,var,freq) |
| 1 | ARHGAP27-Ⅰ | S445L | 11 | 4 | FPFLHFRQFIA | 2 | FPFSHFRQFIA | 2 | HLA-B54:01 | 1960.482039 | 66,43,0.394 | 69,0,0.000 |
| 2 | SLC40A1-Ⅰ | S23F | 10 | 8 | SLADYLTFAK | 14 | SLADYLTSAK | 24 | HLA-A11:01 | 4169 | 47,22,0.319 | 143,0,0.000 |
| 3 | ARHGAP27-Ⅱ | S445L | 18 | 6 | PLFPFLHFRQFIAAIKLQ | 4.04 | PLFPFSHFRQFIAAIKLQ | 5.26 | DRB1:1201 | 1960.482039 | 66,43,0.394 | 69,0,0.000 |
| 4 | SLC40A1-Ⅱ | S23F | 18 | 4 | YLTFAKFLLYLGHSLSTW | 5.75 | YLTSAKFLLYLGHSLSTW | 5.79 | DRB1:1201 | 4169.335764 | 47,22,0.319 | 143,0,0.000 |
|  |  |  |  |  |  |  |  |  |  |  |  |  |
| **patient4** |  |  |  |  |  |  |  |  |  |  |  |  |
| No | gene | amino_acid | length | pos | peptide_mut | affinity_mut(nM) | peptide_wt | affinity_wt(nM) | HLA | tumor_var(rna) | tumor_exome(ref,var,freq) | normal_exome(ref,var,freq) |
| 1 | CHD5-Ⅰ | D799Y | 10 | 6 | EFSFEYNAIR | 44 | EFSFEDNAIR | 156 | HLA-A33:03 | 80 | 269,150,0.358 | 322,0,0.000 |
| 2 | OR10AD1-Ⅰ | P288H | 10 | 4 | MCNHIIYSFR | 16 | MCNPIIYSFR | 15 | HLA-A33:03 | 0 | 77,45,0.369 | 149,0,0.000 |
| 3 | LRFN1-Ⅰ | R151W | 9 | 3 | IRWVESAAF | 213 | IRRVESAAF | 398 | HLA-C07:02 | 0 | 340,183,0.350 | 389,1,0.003 |
| 4 | CHD5-Ⅱ | D799Y | 18 | 1 | YNAIRSGKKVFRMKKEVQ | 15.29 | DNAIRSGKKVFRMKKEVQ | 18.53 | DRB1:1201 | 80 | 269,150,0.358 | 322,0,0.000 |
| 5 | OR10AD1-Ⅱ | P288H | 15 | 4 | MCNHIIYSFRNKEIK | 42.77 | MCNPIIYSFRNKEIK | 90.11 | DRB1:1602 | 0 | 77,45,0.369 | 149,0,0.000 |
| 6 | LRFN1-Ⅱ | R151W | 18 | 12 | RHLILGNNQIRWVESAAF | 34.14 | RHLILGNNQIRRVESAAF | 24.38 | DRB1:1201 | 0 | 340,183,0.350 | 389,1,0.003 |
|  |  |  |  |  |  |  |  |  |  |  |  |  |
|  |  |  |  |  |  |  |  |  |  |  |  |  |
| **patient5** |  |  |  |  |  |  |  |  |  |  |  |  |
| No | gene | amino_acid | length | pos | peptide_mut | affinity_mut(nM) | peptide_wt | affinity_wt(nM) | HLA | tumor_var(rna) | tumor_exome(ref,var,freq) | normal_exome(ref,var,freq) |
| 1 | PIK3CA-Ⅰ | H1047L | 9 | 2 | ALHGGWTTK | 362 | AHHGGWTTK | 13632 | HLA-A11:01 | 54 | 145,28,0.162 | 102,0,0.000 |
| 2 | SLC16A14-Ⅰ | M291R | 11 | 7 | TVSWLTRRVRK | 69 | TVSWLTMRVRK | 42 | HLA-A11:01 | 0 | 108,12,0.100 | 142,0,0.000 |
| 3 | PKP1-Ⅰ | M535L | 9 | 7 | RTYLNLLGK | 15 | RTYLNLMGK | 14 | HLA-A11:01 | 0 | 87,11,0.112 | 147,0,0.000 |
| 4 | SLC16A14-Ⅱ | M291R | 18 | 18 | RKNMCALRILKTVSWLTR | 121.5 | RKNMCALRILKTVSWLTM | 109.6 | DRB1:0405 | 0 | 108,12,0.100 | 142,0,0.000 |
| 5 | PKP1-Ⅱ | M535L | 16 | 8 | IRTYLNLLGKSKKDAT | 116.1 | IRTYLNLMGKSKKDAT | 165.5 | DRB1:0405 | 0 | 87,11,0.112 | 147,0,0.000 |
| 6 | PIK3CA-Ⅱ | H1047L | 18 | 17 | TEQEALEYFMKQMNDALH | 33.5 | TEQEALEYFMKQMNDAHH | 40.8 | DRB1:0405 | 54 | 145,28,0.162 | 102,0,0.000 |
|  |  |  |  |  |  |  |  |  |  |  |  |  |
|  |  |  |  |  |  |  |  |  |  |  |  |  |
| **patient6** |  |  |  |  |  |  |  |  |  |  |  |  |
| No | gene | amino_acid | length | pos | peptide_mut | affinity_mut(nM) | peptide_wt | affinity_wt(nM) | HLA | tumor_var(rna) | tumor_exome(ref,var,freq) | normal_exome(ref,var,freq) |
| 1 | GRM5-Ⅰ | S102L | 11 | 1 | LAVALEQSIEF | 7 | SAVALEQSIEF | 14 | HLA-B35:01 | 8 | 232,110,0.032 | 476,0,0.000 |
| 2 | SPOP-Ⅰ | M117I | 11 | 5 | ETKAIESQRAY | 11 | ETKAMESQRAY | 22 | HLA-A26:01 | 59 | 82,60,0.423 | 120,0,0.000 |
| 3 | KRAS①-Ⅰ | G12V | 8 | 6 | VVVGAVGV | 16 | VVVGAGGV | 52 | HLA-A02:06 | 101 | 11,13,0.542 | 61,0,0.000 |
| 4 | MCL-6-Ⅰ | E141K | 10 | 8 | SAIKPPRKEF | 183 | SAIKPPREEF | 78 | HLA-C03:04 | 11 | 128,64,0.333 | 138,0,0.000 |
| 5 | KRAS②-Ⅰ | G12V | 8 | 8 | KLVVVGAV | 41 | KLVVVGAG | 6783 | HLA-A02:06 | 101 | 11,13,0.542 | 61,0,0,0.000 |
|  |  |  |  |  |  |  |  |  |  |  |  |  |
| **patient7** |  |  |  |  |  |  |  |  |  |  |  |  |
| No | gene | amino_acid | length | pos | peptide_mut | affinity_mut(nM) | peptide_wt | affinity_wt(nM) | HLA | tumor_var(rna) | tumor_exome(ref,var,freq) | normal_exome(ref,var,freq) |
| 1 | ARHGAP12-Ⅰ | G495V | 11 | 7 | SWAVLQVSSLL | 104 | SWAVLQGSSLL | 122 | HLA-C14:03 | 105 | 120,8,0.062 | 142,0,0.000 |
| 2 | PRRC2C-Ⅰ | S2260Y | 11 | 1 | YPNVREKGSPV | 58 | SPNVREKGSPV | 927 | HLA-B54:01 | 63 | 121,9,0.069 | 128,0,0.000 |
| 3 | ETV6-Ⅰ | V345I | 9 | 6 | LLWDYIYQL | 2 | LLWDYVYQL | 2 | HLA-A02:01 | 0 | 65,6,0.085 | 95,0,0.000 |
| 4 | HOXA5-Ⅰ | F5I | 8 | 1 | IVNSFCGR | 21 | FVNSFCGR | 15 | HLA-A33:03 | 0 | 113,12,0.096 | 131,0,0.000 |
| 5 | MUC16-Ⅰ | P14341L | 11 | 10 | LPYSQDKAQLG | 143 | LPYSQDKAQPG | 60 | HLA-B54:01 | 0 | 73,4,0.052 | 148,0,0.000 |
| 6 | ARHGAP12-Ⅱ | G495V | 18 | 17 | GKKVRKNWLSSWAVLQVS | 49 | GKKVRKNWLSSWAVLQGS | 63 | DRB1:1501 | 105 | 120,8,0.062 | 142,0,0.000 |
| 7 | PRRC2C-Ⅱ | S2260Y | 18 | 1 | YPNVREKGSPVTSTAPPI | 71.8 | SPNVREKGSPVTSTAPPI | 71.9 | DRB1:1302 | 63 | 121,9,0.069 | 128,0,0.000 |
| 8 | ETV6-Ⅱ | V345I | 18 | 14 | IGRIADCRLLWDYIYQLL | 33.8 | IGRIADCRLLWDYVYQLL | 34.8 | DRB1:1501 | 0 | 65,6,0.085 | 95,0,0.000 |
| 9 | HOXA5-Ⅱ | F5I | 16 | 5 | MSSYIVNSFCGRYPNG | 334.1 | MSSYFVNSFCGRYPNG | 1306.7 | DRB1:1302 | 0 | 113,12,0.096 | 131,0,0.000 |
