# Supplementary material for: Dendritic cell-derived exosomes induce monocyte antigen-presentation and immune amplification in neoantigen vaccine therapy
Source: Front Immunol. 2025 May 19;16:1565696. doi: 10.3389/fimmu.2025.1565696 (PMC12127191; doi:10.3389/fimmu.2025.1565696)
Supplement: Supplementary file 2 [file Table2.docx]

Additional experiments

Mo-CD14　1X10^4cells/well
Post- treatment CD4+ or CD8+ lymphocytes 1X10^5cells/well
Spot detection after 72hours

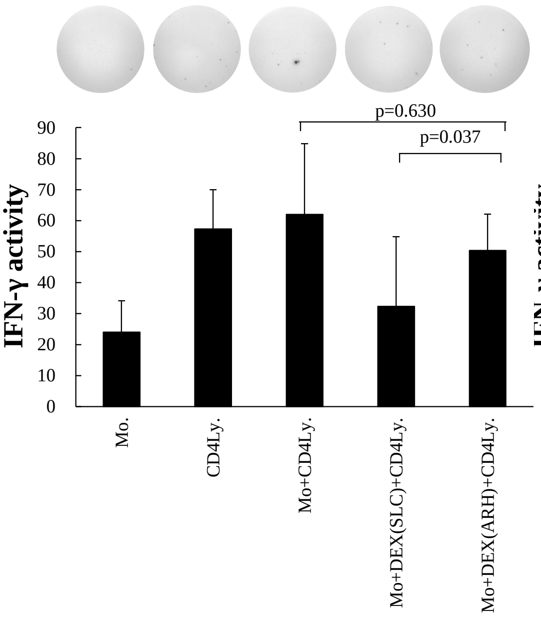

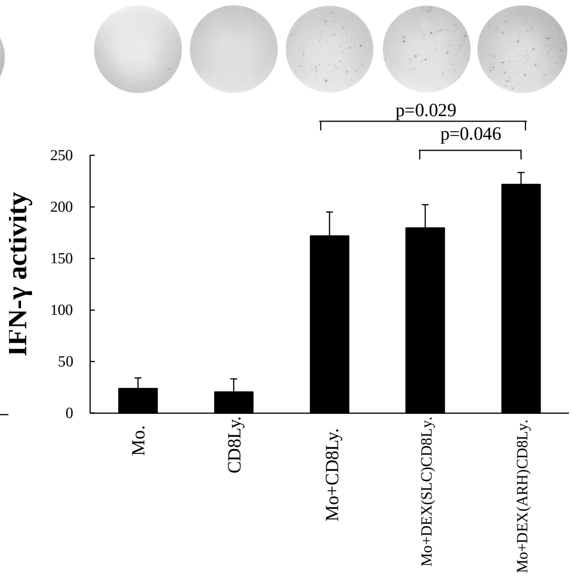

SLC：SLC40A1-Ⅱ（control neoantigen）
ARH：ARHGAP27-Ⅱ（Neoantigen that showed an immunological response）
